# Photodynamic Antibacterial Nanofibers with Tunable Pro- and Antioxidant Activity via N,S-Doped Carbon Quantum Dots for Corneal Tissue Engineering

**DOI:** 10.1021/acsami.5c16701

**Published:** 2025-12-09

**Authors:** Roksana Kurpanik, Anna Ścisłowska-Czarnecka, Zofia Kucia, Agnieszka Lechowska-Liszka, Nikola Lenar, Agnieszka Różycka, Marcin Sarewicz, Grzegorz Szewczyk, Ewa Stodolak-Zych

**Affiliations:** † Department of Biomaterials and Composites, Faculty of Materials Science and Ceramics, 49811 AGH University of Krakow, Krakow 30-059, Poland; ‡ Department of Applied Cosmetology, University of Physical Education, Krakow 31-571, Poland; § Department of Silicate Chemistry and Macromolecular Compounds, Faculty of Materials Science and Ceramics, AGH University of Krakow, Krakow 30-059, Poland; ∥ Department of Analytical Chemistry and Biochemistry, Faculty of Materials Science and Ceramics, AGH University of Krakow, Krakow 30-059, Poland; ⊥ Department of Building Materials Technology, Faculty of Materials Science and Ceramics, AGH University of Krakow, Krakow 30-059, Poland; # Department of Biophysics, Faculty of Biochemistry, Biophysics and Biotechnology, 98817 Jagiellonian University, Krakow 31-387, Poland; ¶ Department of Molecular Biophysics, Faculty of Biochemistry, Biophysics and Biotechnology, 98817 Jagiellonian University, Krakow 31-387, Poland

**Keywords:** carbon quantum dots, antibacterial photodynamic inactivation, core−shell nanofibers, cornea regeneration, redox-active biomaterials

## Abstract

Antibiotic resistance poses a critical challenge in ocular medicine, where treatments must combine antibacterial potency with tissue compatibility. Electrospun core–shell nanofibers offer an attractive solution for ocular applications as they provide a biomimetic extracellular matrix structure with controlled drug release and surface functionality. In this work, polycaprolactone (PCL) was used as the mechanically robust, biodegradable core, while polyvinylpyrrolidone (PVP) formed a hydrophilic shell to enhance wettability and ocular compatibility. The nanofibers were further functionalized with N,S-doped carbon quantum dots, exhibiting light-switchable redox behavior. Compositional and spectroscopic analyses revealed that N,S-doped CQDs possessed a significantly narrowed bandgap (3.14 eV) relative to cysteine- and tryptophan-derived CQDs, attributable to heteroatom-induced defect states and the formation of π–π conjugated domains, confirmed by X-ray photoelectron spectroscopy (XPS) and Fourier transform infrared spectroscopy (FT-IR). XPS measurements showed valence band energies suitable for superoxide generation under illumination, consistent with the reported redox potentials. As a result, illuminated nanofibers produced reactive oxygen species (ROS), reducing *Escherichia coli* and *Staphylococcus aureus* populations by 90% and 80%, respectively. In the dark, the same CQDs exhibited up to 90% radical-scavenging activity, increasing BJ human fibroblast viability by 35%. Additional mechanistic evidence indicated that light enhances the adhesion of CQDs to bacterial membranes, further promoting ROS-mediated inactivation. With a high quantum yield of 50% and strong blue fluorescence (λ_em_ = 445 nm, λ_ex_ = 380 nm), the CQDs also offer imaging and diagnostic potential. Together, these findings position N,S-doped CQDs-modified core–shell nanofibers as a biologically adaptive platform capable of photodynamic antibacterial action while supporting cytoprotection and tissue regeneration—an innovative approach for combating antibiotic-resistant ocular infections.

## Graphical abstract

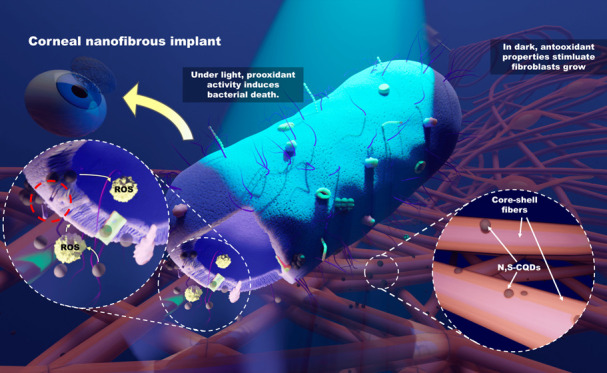

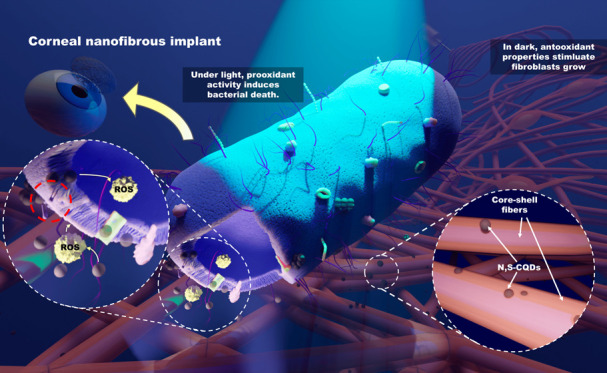

## 1 Introduction

The growing global threat of antimicrobial resistance (AMR) has prompted an urgent search for innovative and effective strategies to combat pathogenic microorganisms. The overuse of conventional antibiotics, ineffective infection control in healthcare settings, and inadequate sanitation practices have led to the emergence of multidrug-resistant bugs (MDRs), rendering many traditional treatments ineffective.^1^ Given that the eye, particularly the cornea, is a highly exposed and sensitive tissue, it is especially vulnerable to microbial invasion. Corneal infections, particularly bacterial keratitis, are one of the main causes of vision loss worldwide. Over 90% of microbial keratitis cases are bacterial in origin. This can lead to corneal scarring, stromal melting, and perforation and accounts for at least 1.5 million cases of unilateral blindness each year.^2,3^ The growing prevalence of multidrug-resistant ocular pathogens severely limits the success of traditional antibiotic therapy, emphasizing the urgent need for alternative, locally effective, and biocompatible antimicrobial strategies.

Tissue engineering and biomaterial-based therapies have emerged as promising alternatives for addressing both infection control and tissue regeneration in the cornea. Among various biomaterials, electrospun fibrous scaffolds are particularly attractive due to their high surface area, tunable porosity, and ability to mimic the native extracellular matrix (ECM), thereby supporting cell adhesion, migration, and differentiation.^4^ Within this class, core–shell nanofibers have gained increasing attention because of their ability to combine two complementary polymer systems: a mechanically robust, biodegradable core and a hydrophilic, biocompatible shell. Polycaprolactone (PCL) is widely used as a core material due to its strength and biodegradability, while polyvinylpyrrolidone (PVP) serves as a hydrophilic shell that enhances surface wettability and ocular compatibility. This architecture also enables the functionalization of the fiber surface with therapeutic nanomaterials, making it an ideal platform for corneal regeneration.^5^

Despite these advantages, the incorporation of antimicrobial agents into scaffolds remains challenging. Various antibacterial agents, including metal nanoparticles (e.g., silver, gold), polyphenols, and antibiotics, have been incorporated into nanofibers; nevertheless, their biomedical application is hindered by rapid in vivo degradation and the risk of hemolytic and immunogenic reactions.^6–11^ Hence, the development of nontoxic, stimuli-responsive antibacterial systems capable of precise temporal control over antimicrobial activity is a crucial step toward safer, multifunctional ocular materials.

In this context, carbon quantum dots (CQDs) have emerged as a new class of light-responsive nanomaterials that combine strong photoluminescence, good biocompatibility, and ease of functionalization with high photostability and strong ROS-generating potential.^12^ Their ability to generate reactive oxygen species is closely linked to their graphitic core, surface chemistry (presence of oxygen-containing groups), and photophysical properties. This ability can be enhanced by introducing structural defects and energy states through doping with heteroatoms, particularly nitrogen and sulfur. These dopants increase the photosensitivity and quantum yield of CQDs.^13,14^ Such properties have sparked widespread interest in the use of CQDs as photosensitizers in antibacterial photodynamic inactivation (aPDI). Carbon quantum dots can originate from a wide range of sources, including organic (natural and synthetic) and inorganic materials. Among these, amino acids have attracted significant research interest due to their natural abundance, biocompatibility, and inherent heteroatom content.^15^ Recent studies have demonstrated the potential of amino acid-derived CQDs as efficient photosensitizers for aPDI. Kang et al. showed that heteroatom doping originating from amino acid precursors significantly enhances visible-light-driven antibacterial activity by promoting ROS generation and improving charge separation efficiency.^16^ Similarly, Nie et al. embedded CQDs within electrospun polyacrylonitrile nanofibers to create a light-responsive composite capable of producing singlet oxygen under visible light, achieving over 99% bacterial inactivation.^17^ Milenković et al. and Suner et al. reported that amino acid-modified or arginine-derived CQDs exhibit strong blue-light photoactivity, leading to oxidative bacterial membrane damage through photodynamically generated ROS.^18^ Collectively, these studies establish amino acid-derived CQDs as promising, naturally sourced nanophotosensitizers for light-activated antibacterial applications.

Although recent studies have demonstrated the antimicrobial potential of amino acid-derived CQDs through light-induced ROS generation, their applications have largely remained confined to *in vitro* antibacterial assessments with limited understanding of the underlying electronic and redox mechanisms. Most reports focus on demonstrating photodynamic efficacy under illumination, yet they overlook the influence of dopant chemistry on band structure, charge carrier dynamics, and redox reversibility factors that critically determine both the magnitude and controllability of ROS production. Furthermore, these studies rarely examine the behavior of CQDs under dark conditions, leaving their antioxidant or cytoprotective capabilities unexplored. Equally underinvestigated is the integration of amino acid-derived CQDs into biocompatible fibrous scaffolds, which could enable localized, sustained, and responsive antibacterial functionality suitable for delicate tissues such as the cornea. Furthermore, the precise interactions between CQDs and bacterial membranes have yet to be fully elucidated. These interactions are governed by factors such as nanoparticle size, surface charge (zeta potential), and the chemical composition of bacterial outer membranes. Zeta potential analysis represents a valuable yet underutilized tool for probing these electrostatic and mechanical interactions, offering insight into the mechanisms that govern bacterial adhesion and cell wall disruption.^19^

To address these knowledge gaps, this study presents electrospun PCL/PVP core–shell nanofibers functionalized with N,S-doped carbon quantum dots. The platform is designed to combine mechanical robustness, biocompatibility, and light-activated antibacterial activity with a dark-state antioxidant functionality. In particular, we investigate how N and S dopants modulate charge separation, ROS generation, and antibacterial efficiency, providing mechanistic insight into the photodynamic processes. This approach establishes a direct link between the dopant-induced electronic structure, redox behavior, and biological performance, offering a strategy for designing safe and effective ocular biomaterials for infection control and tissue regeneration.

## 2 Materials and Methods

### 2.1 Materials

Polycaprolactone (PCL) pellets (M.W. = 80 kDa), l-cysteine (M.W. = 121.16 Da), l-tryptophan (M.W. = 204.23 Da), phosphate buffer solution (PBS), Triton X-100, and 2′,7′-dichlorofluorescein diacetate (DCFH-DA) were purchased from Sigma-Aldrich (Merck, Germany). Polyvinylpyrrolidone (PVP) powder (M.W. = 1300 kDa) was purchased from Acros Organics (Belgium). Dichloromethane (DCM), dimethylformamide (DMF), ethanol (99.8% CZDA), and dimethyl sulfoxide (DMSO) were purchased from POCH (Avante Performance Materials S.A, Poland). *Escherichia coli* (ATCC 8739) and *Staphylococcus aureus* strains (ATCC 6538P) as well as tryptic soy broth (TSB), tryptic soy agar (TSA), plate count agar (PCA), and Mueller–Hinton agar (MHA) were purchased from BioMaxima (Poland). BJ CRL-2522 human fibroblasts were purchased from the American Type Culture Collection (ATCC; Manassas, VA, USA). ViaLightPlus and ToxiLightPlus tests for biological studies were purchased from Lonza (BioAssay Kit, Lonza, Switzerland). Sterilized deionized water was used throughout all experiments.

### 2.2 Synthesis of Carbon Quantum Dots

Carbon quantum dots were synthesized by a one-pot hydrothermal method (see Figure 1a). l-Cysteine and l-tryptophan were used as the CQD precursors with a molar ratio of 1:1. In brief, 0.121 g of l-cysteine and 0.204 g of l-tryptophan were suspended in 15 mL of deionized water and homogenized with ultrasounds Then, the solution was transferred to a Teflon-sealed autoclave and heated at 160 °C for 6 h. After cooling down to room temperature, the solution was filtered through a 0.22 μm cellulose acetate filter, dialyzed against a 1 kDa cellulose membrane for 24 h, and freeze-dried. For bacterial testing, four concentrations of CQDs were prepared (Table 1).

**Figure 1:**
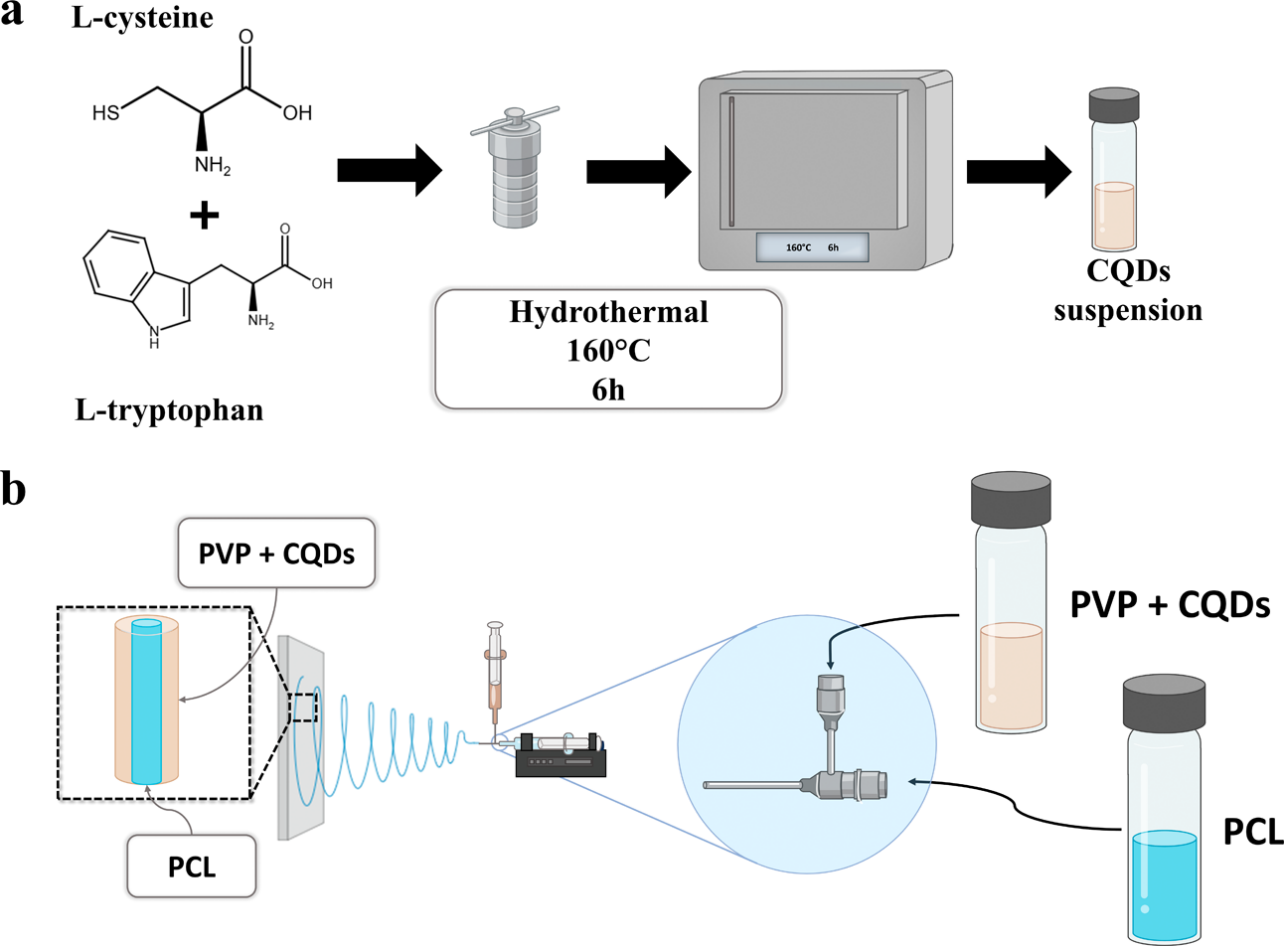
Scheme of the CQDs hydrothermal synthesis (a) as well as the scheme of electrospinning of CQDs-modified core–shell fibers (b).

**Table 1:** Description and Concentration of the Tested Samples

Symbol	Concentration(mg/mL)
CQDs 1	1.000
CQDs 1/2	0.500
CQDs 1/4	0.250
CQDs 1/8	0.125

### 2.3 Electrospinning of Core–Shell Fibers

The core–shell fibers were obtained by the coaxial electrospinning method (see Figure 1b). The core of nanofibers was produced from a 10 wt % solution of PCL in a (1:1 v/v) mixture of DCM and DMF. The shell layer consisted of an 8 wt % solution of PVP dissolved in a solvent system of ethanol and DMF (1:1 v/v). The electrospinning process was carried out at ambient temperature (25 °C) and relative humidity (20%). The spinning solutions were supplied separately to the coaxial needle at a flow rate of 0.5 mL/h (PVP) and 0.2 mL/h (PCL). The voltage was set at 18 kV. The fibers were collected on a drum collector rotating at 300 rpm and positioned at a distance of 13 cm from the needle.

The CQDs-modified nanofibrous scaffold was prepared by dispersing 40 mg of the CQDs in 5 mL of PVP solution, followed by ultrasound homogenization. The electrospinning parameters remained the same.

### 2.4 Characterization of CQDs

The morphology of synthesized CQDs was assessed using high-resolution transmission electron microscopy (HR-TEM) (JEM-ARM200F NEOARMex, JEOL, Japan) and atomic force microscopy (AFM). For TEM observations, samples were drop-cast onto a copper TEM grid and left to dry in air. Based on the obtained images, the average size of the CQDs was determined by measuring the diameter of 100 random carbon quantum dots. The AFM images were taken using a MultiMode VIII microscope (Bruker, USA) equipped with an antimony-doped silicon probe with the following parameters: a nominal tip radius equal to 8 nm and a nominal spring constant of 40 N/m. A semicontact (tapping) mode was utilized for the measurements.

The structure of the obtained CQDs was analyzed using FT-IR spectroscopy in the ATR mode using a Tensor 27 FT-IR spectrophotometer (Bruker, USA). Infrared spectra were acquired for 256 scans in the range of 4000–400 cm^–1^ and spectral resolution of 4 cm^–1^. XPS measurements were performed with a PHI VersaProbe II Scanning XPS apparatus. The samples were irradiated with a focused monochromatic Al Kα (E = 1486.6 eV) X-ray beam with a diameter of 100 μm, and the beam was rastered over an area of 400 × 400 μm^2^. The pass energy of the analyzer was set to 117.50 eV for survey scans and 46.95 eV to obtain high-resolution spectra. A double neutralization with low-energy monatomic Ar^+^ ions and 1 keV electrons was used to avoid charging effects. The spectra were referenced to the neutral (C–C) carbon C 1 s peak, with a binding energy of 285.0 eV. The operating pressure in the analytical chamber was less than 3 × 10^–9^ mbar. Deconvolution of spectra was carried out using PHI MultiPak software (v.9.9.3). Spectrum background was subtracted using the Shirley method. Raman spectroscopy was performed using 488 and 785 nm lasers on an Alpha 300 M+ spectrometer (WITec, Germany). An area of 20 × 20 μm was analyzed with a 1 μm step. For the 785 nm laser, the power was set to 7 mW, while a 10 s integration time for each measurement was used along with a 300 grating and Zeiss 50× objective. For the 488 nm imaging, the laser power was set to 0.2 mW and the integration time to 0.3 s. In addition, 600 gratings and a 50× objective were used. Raman images were generated as the sum (integral intensities) of bands—for 785 nm, it was 1009 cm^–1^ and for the 488 nm emission band at approximately 510 nm.

The zeta potential of the CQDs was measured with a Zetasizer NanoZS apparatus (Malvern Instr., UK) equipped with folded capillary cells. A suspension of CQDs in PBS with a concentration of 0.1 wt % and a neutral pH (∼7.1) was prepared for the study. Measurements were conducted at 25 °C after waiting two min for the system to stabilize.

The optical properties of the CQDs were assessed using UV–Vis and fluorescence microscopy. Both spectra were recorded on an aqueous suspension of the CQDs. UV–Vis absorbance spectra were recorded using a UV-2600i spectrophotometer (SHIMADZU, Japan). An FP-8500 spectrofluorometer (Jasco, UK) was used to obtain the fluorescence spectra and fluorescence quantum yield. The fluorescence quantum yield (Φ*F*) of the CQDs was determined using Rhodamine B dissolved in H_2_O (the concentration was adjusted to obtain an absorbance value below 1.0) as the reference standard. The Φ*F* (%) was calculated using (eq 1):

1 $$ØF_{U\;}\left(\%\right)=ØF_{S}\times F_{U}F_{S}\times A_{S}A_{U}\times 100$$

where Φ*F*_U_ is the fluorescence quantum yield of the sample, Φ*F*_S_ is the fluorescence quantum yield of the reference standard, *F*_U_ is the fluorescence intensity at an excitation wavelength of 380 nm for the sample, *F*_S_ is the fluorescence intensity at an excitation wavelength of 380 nm for the reference standard, *A*_U_ is the absorbance value at OD_380_ for the sample, and *A*_s_ is the absorbance value at OD_380_ for the reference standard.

To determine the impact of heteroatom doping on optical activity and, thus, the ROS generation efficiency, the optical bandgap energy (*E*_g_) of the CQDs was calculated using the Tauc relation (eq 2):

2 $$\alpha h\nu =A{(h\nu -E_{g})}^{n}$$

where *A* is a constant, α is the absorption coefficient, *h* is Planck’s constant, *ν* is the photon frequency, and *n* represents the transition type (*n* = 1/2 for indirect and n = 2 for direct transitions). For CQDs, the indirect and direct transition models were applied, and (α*hν*)^*n*^ versus *hν* plots were constructed from UV–Vis absorption spectra. The bandgap energy (*E*_g_) was determined by extrapolating the linear portion of the curve to the photon energy axis.

### 2.5 Characterization of CQDs-Modified Nanofibers

The microstructure of the obtained fibrous substrates was observed using an Apreo 2 scanning electron microscope (Thermo Fisher Scientific, USA). Based on the obtained images, the diameters of 100 randomly selected fibers were measured.

Encapsulation efficiency (EE) and loading efficiency (LE) were determined by using fluorescence measurement. To calculate the concentration of released carbon quantum dots, standard curves with known CQDs concentrations (0.2, 0.4, 0.6, 0.8, and 1 mg/mL) were used. To determine the CQDs concentration in the PVP layer (fibers’ shell), three 16 mm diameter samples (0.5 mg) were cut from the nonwoven material and washed with a DMF solution. The resulting suspension of PVP and CQDs was combined with water and then centrifuged at 5000 rpm. After that, 200 μm of supernatant was taken and placed in a black 96-well plate. The experiments were conducted on a FluoStar Omega fluorometer (BMG Labtech, Germany) at λ_ex_ = 370 nm and λ_em_ = 450 nm.

The loading (eq 3) and encapsulation (eq 4eq 4) efficiencies were calculated according to the formulas:

3 $$\%LE=\frac{mass\;of\;CQDs\;\mathrm{in}\;nonwoven}{mass\;of\;nonwoven}\times 100$$

4 $$\%EE=\frac{mass\;of\;CQDs\;\mathrm{in}\;nonwoven}{mass\;of\;CQDs\;\mathrm{in}itia\mathrm{ll}y\;added\;to\;the\;spinning\;solution}\times 100$$

### 2.6 Antibacterial Assays

#### 2.6.1 Sample Preparation

Prior to the antibacterial studies, the minimum inhibitory concentration (MIC) of CQDs was determined. The bacterial strains were cultured overnight in nutrient broth (NB) at 37 °C, centrifuged at 5000 rpm for 5 min, and resuspended in Mueller–Hinton (MH) broth. The bacterial suspension was adjusted to the 0.5 McFarland standard using a turbidimeter and subsequently 2-fold diluted to obtain a final concentration of approximately 1 × 10^6^ CFU/mL. Next, 100 μL of the bacterial suspension was inoculated into a 96-well microtiter plate containing serial dilutions of CQDs (5.0, 2.5, 1.25, 0.625, and 0.3125 mg/mL). The samples were then exposed to LED light (390–700 nm), and absorbance was measured every hour over an 8-h period. The MIC was defined as the lowest CQDs concentration that completely inhibited visible bacterial growth. For the aPDI studies, the CQDs powder was dispersed in PBS to obtain a 1 mg/mL stock solution. To verify the impact of the CQDs concentration on antibacterial efficacy, all experiments were conducted using a series of dilutions of the stock solution in ranges of 1 mg/mL, 0.5 mg/mL, 0.25 mg/mL, and 0.125 mg/mL. The prepared suspensions were stored at 4 °C in the dark.

The CQDs-modified nonwoven was cut into 16 mm disks, sterilized by UV irradiation, and placed in a 24-well plate for further studies.

#### 2.6.2 Bacteria Culture

Prior to the experiment, both strains (*S. aureus* and *E. coli*) were streaked from a −40 °C stored freezer on TSA and incubated for 24 h at 37 °C. Then, one colony from each strain was transferred to 5 mL of TSB and cultured overnight at 37 °C. Then, an aliquot of 5 mL of the overnight incubated strains was centrifuged at 5000 rpm for 10 min. The supernatant containing TSB was discarded, and the pelleted cultures were resuspended in sterile PBS. The strain concentration was adjusted to 0.5 McFarland (1.5 × 10^8^ colony-forming units (CFU/mL)) using a densitometer.

#### 2.6.3 Disk–Diffusion Test

The impact of the non-illuminated CQDs on both bacterial strains was evaluated using the Kirby–Bauer test. A bacterial inoculum containing 0.5 McFarland of bacterial suspension in saline was spread on the MHA with a sterile swab. Then, 50 μL of each CQDs concentration (CQDs 1 – CQDs 1/8) was dropped on the surface of the MHA and incubated overnight at 37 °C. After that, it was checked if any zones of inhibition appeared around the applied samples.

#### 2.6.4 Antibacterial Photodynamic Inactivation Assay

During the aPDI test, all bacterial strains were illuminated with a diode lamp (30 W) at a distance of 5 cm. The light spectrum of the lamp was within a range of 400–780 nm.

The samples for the aPDI test were divided into four groups (see Figure 2): dark control (bacteria in the dark), light control (illuminated bacteria), dark sample (the CQDs/nonwoven with bacteria in the dark), and light sample (illuminated CQDs/nonwoven with bacteria). For the dark and light control groups, 1 mL of 0.5 McFarland bacterial suspension in PBS was added to 24-well plates. For CQD samples, 100 μL of solution from each concentration (1, 0.5, 0.25, and 0.125 mg/mL) was added to 1 mL of bacterial suspension and incubated for 1 h at 37 °C in the dark. After incubation, light groups were illuminated for a period of 1, 2, 3, and 4 h. The dark groups were wrapped in aluminum foil and incubated for the same period of time. After photosensitization, the bacterial samples were taken from the wells, and a 10-fold serial dilution in PBS was performed. Then, 100 μL of each dilution was spread on plate count agar and incubated for 24 h at 37 °C. After incubation, the number of viable bacteria was counted according to eq 5:^20^

5 $$\frac{CFU}{ml}=\frac{number\;of\;colonies\times dilution\;factor}{volume\;of\;culture\;plated}$$

**Figure 2:**
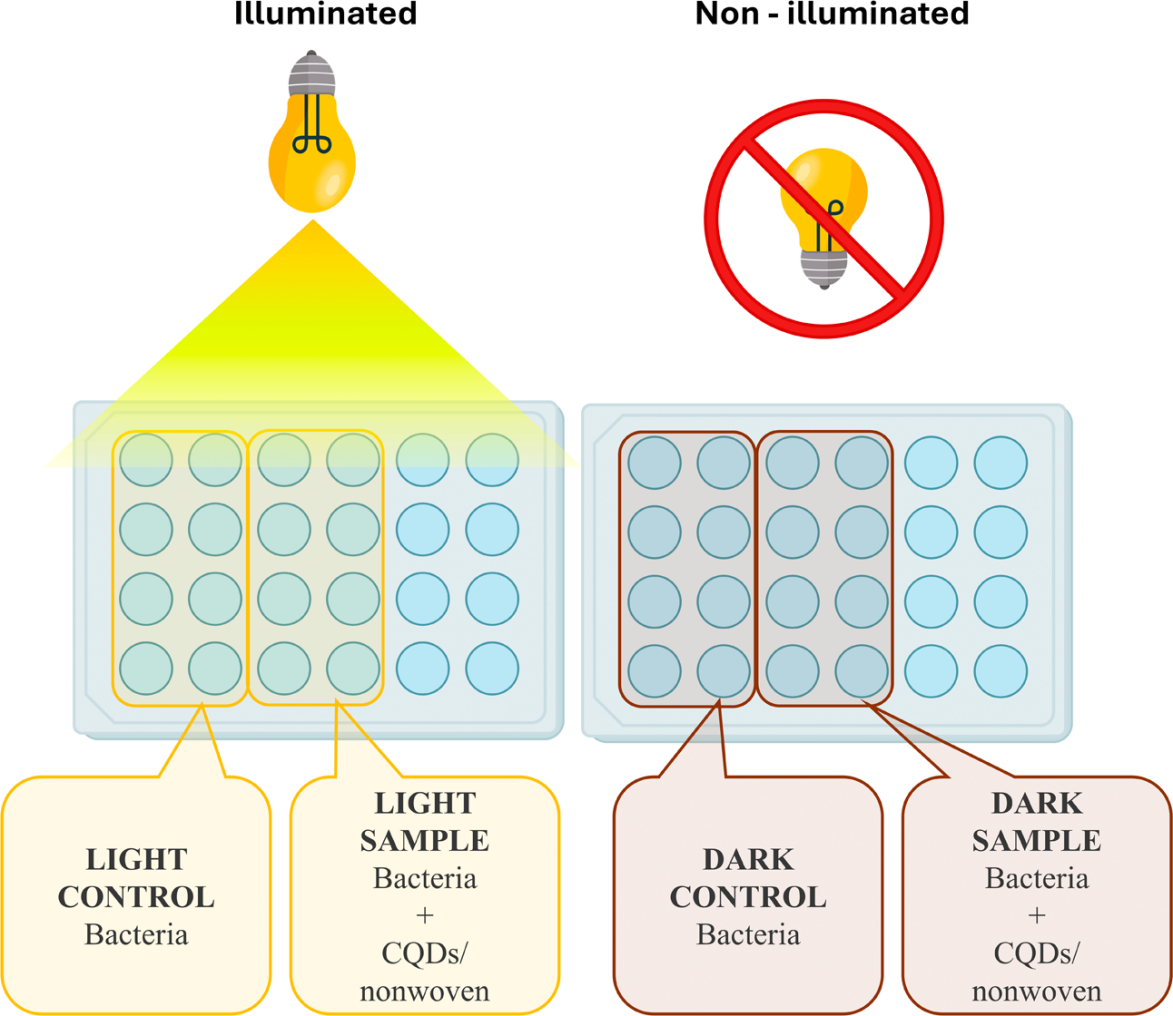
Scheme of the aPDI experiment and testing groups.

#### 2.6.5 Bacterial Membrane Permeability

The outer membrane permeability of *E. coli* and *S. aureus* strains was evaluated using *N*-phenyl-1-naphthylamine (NPN) as a fluorescent probe, following the method of Qin et al. with minor modifications.^21^ Bacterial suspensions (1 × 10^6^ CFU/mL) in PBS were treated with CQDs (illuminated and non-illuminated) for 1 h and then mixed with 200 μM NPN to a final volume of 300 μL. Fluorescence was recorded at λ_ex_ = 350 nm and λ_em_ = 420 nm using a FluoStar Omega plate reader (BMG Labtech, Germany). An increase in fluorescence intensity indicated enhanced outer membrane permeability.

#### 2.6.6 Endogenous ROS Detection

DCFH-DA, a cell-permeable reagent, was used as the indicator for ROS, as it is rapidly oxidized to highly fluorescent 2′,7′-dichlorodihydrofluorescein (DCF) by ROS. To quantify endogenous ROS, *E. coli* and *S. aureus* strains were inoculated together with CQDs. After exposure, the strains were incubated with a 10 μM DCFH-DA solution in the dark at 37 °C for 30 min. The stained cells were then placed in the plate reader to measure the fluorescence at λ_ex_ = 495 nm and λ_em_ = 517 nm. Electron paramagnetic resonance (EPR) spin-trapping experiments were performed using 5,5-dimethyl-1-pyrroline *N*-oxide (DMPO, 100 mM) as the spin-trapping agent. Samples containing DMPO and CQDs suspension (approximately 25 μg/mL) in 99% DMSO were placed in 0.3 mm-thick quartz EPR flat cells and irradiated *in situ* within the resonant cavity using 370 nm LED light. EPR spectra were recorded on a Bruker EMX-AA spectrometer (Bruker BioSpin, Germany) under the following conditions: center field, 3388 G; sweep width, 25 G; attenuation, 13 dB; microwave power, 10.6 mW; modulation amplitude, 0.5 G; time constant, 0.229 s; sweep time, 82 s; and 60 independent scans per measurement series. Simulation of the EPR spectra was carried out by using the EasySpin toolbox for MATLAB, employing standard spectral fitting procedures to analyze radical adduct formation.

#### 2.6.7 Antioxidant Properties

The antioxidant capacity of the additives was evaluated by using the DPPH protocol. For this purpose, a solution of 0.1 mmol/L DPPH (2,2-diphenyl-1-picrylhydrazyl) in ethanol was prepared and homogenized in an ultrasonic bath for 30 s. Samples of the test materials were dissolved in water. Then, 2 mL was taken from each of them and added to 2 mL of DPPH solution. The whole mixture was vigorously stirred and incubated in the dark, at room temperature, for 30 min. In addition, reference solutions were prepared, where 2 mL of the DPPH solution was replaced by 2 mL of ethanol, and a blank solution, where the samples were completely replaced with ethanol. Absorbance measurements of each solution were carried out at a wavelength of 517 nm by using a UV-2600i spectrophotometer (SHIMADZU, Japan). Free radical scavenging activity (SA-scavenging activity) was calculated according to eq 6:^14^

6 $$SA\left[\%\right]=\frac{1-\left(A_{S}-A_{C}\right)}{A_{0}}$$

where *A*_S_–absorbance of the sample, *A*_C_–absorbance of the control (the DPPH solution replaced by ethanol), and *A*_0_–absorbance of the blank (the sample replaced by ethanol).

### 2.7 *In Vitro* Studies

The effect of the nonwovens on the viability of human fibroblasts BJ was evaluated by quantitative methods using the ViaLight and ToxiLight protocols. Prior to the *in vitro* testing, samples in the form of 16 mm diameter discs were cut from the nonwovens, sterilized using UV light (15 min on each side), and then placed in a 24-well plate. Then, the fibroblasts were added to the samples in an amount of 10000 cells per well. The samples were incubated at 37 ± 1 °C and a CO_2_ concentration of 5% for a period of 7 days. Viability and cytotoxicity were measured after the third and seventh days of incubation according to the protocol^22,23^ using a FluoStar Omega luminometer (BMG Labtech, Germany). Samples for fluorescent microscope imaging were stained with a solution of 4′,6-diamidino-2-phenylindole (DAPI, Thermo Fisher, USA) and Alexa Fluor 488-Phalloidin (Thermo Fisher, USA) to visualize their nucleus and F-actin component, respectively. Briefly, the cells were washed with PBS and incubated with a 1% bovine serum albumin solution for 30 min. After that time, the cells were washed with PBS again and fixed with 250 μL of 4% paraformaldehyde for 10 min. Then, the cells were treated with 0.1% Triton X-100 solution for 5 min, washed with PBS, and incubated in the dark for 2 h with Phalloidin. Then, DAPI was added to the cells for 5 min. The samples were observed under an AxioVert Inverted LED fluorescence microscope (Zeiss, Germany).

### 2.8 Statistical Analysis

Each experiment in the study was conducted in triplicate unless otherwise specified. The results are presented as the arithmetic mean ± standard deviation (SD). The level of significance in the biological tests was determined using two-way analysis of variance (ANOVA) followed by Tukey’s post hoc analysis (Origin 2023b software). The results were considered statistically significant when probability values were less than 0.05, unless otherwise specified.

## 3 Results and Discussion

### 3.1 Characterization of CQDs

The HR-TEM image of CQDs is presented in Figure 3. The obtained dots exhibited a spherical shape (also shown in the AFM image in Figure 3e) and an unimodal, rightward-skewed size distribution in the range of 7–20 nm (average size 10 nm ± 2.39). The HR-TEM images (see Figure 3b–d) revealed the presence of both crystalline (see Figure 3b,c) and amorphous phases (see Figure 3d). To show the crystalline structure and calculate the lattice parameters of the CQDs, HR-TEM images were filtered by the FFT (fast Fourier transform), threshold adjustment, and inverted FFT (the zoomed-in pictures in Figure 3b,c). The lattice fringes in the photos of crystalline CQDs correspond to a d-spacing of 0.21 nm (see Figure 3b) and 0.33 nm (see Figure 3c), matching the in-plane (100) and interlayer (002) spacing of graphite, respectively.^14^ Moreover, the FFT image showed the hexagonal structure in the (001) plane, proving the graphitic framework of the obtained CQDs. On the other hand, the FFT image shown in Figure 3d confirmed the presence of an amorphous phase in the CQD structure. However, instead of an axially symmetrical diffraction halo characteristic of a fully amorphous structure, two arcs with strong intensity were present. This can be associated with the presence of a crystalline core surrounded by an amorphous shell. Such a core–shell structure is characteristic of CQDs.^24^

**Figure 3:**
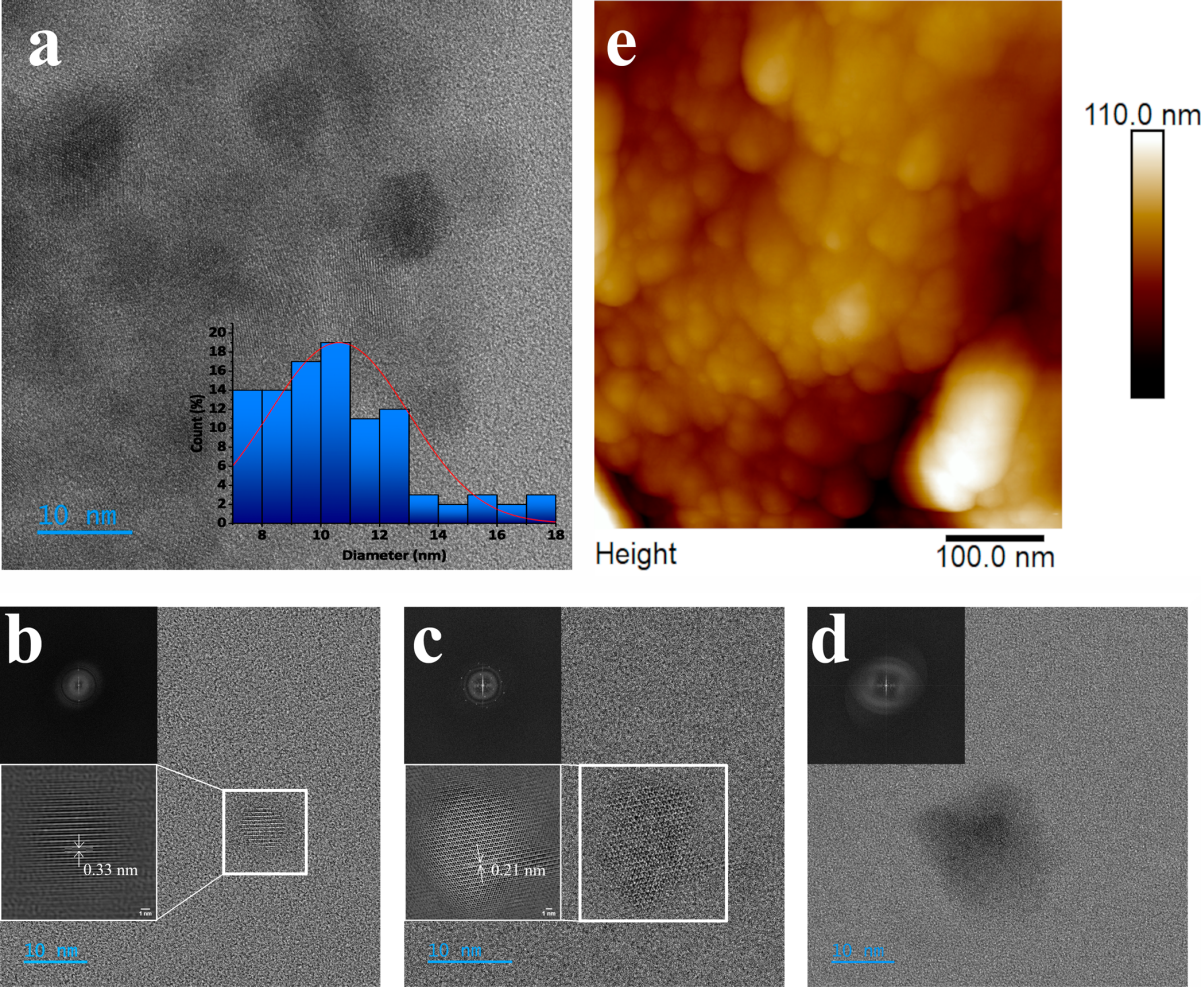
TEM image of the sample with the size distribution of the CQDs (a); HR-TEM images of the crystalline (b–interlayer spacing, c–in-plane spacing) and partially crystalline (d) CQDs; inset: FFT from HR-TEM image; AFM image of the CQDs (e).

The FT-IR spectra of CQDs and both precursors (l-cysteine and l-tryptophan) are shown in Figure 4. In the spectrum of pure l-tryptophan, strong absorption bands present at 3404 cm^–1^ and 1589 cm^–1^ are associated with N–H stretching vibrations of the indole ring and bending in amines, respectively. The bands at 3038 cm^–1^, 2561 cm^–1^, 1666 cm^–1^, 1414 cm^–1^, and 1356 cm^–1^ are associated with unsaturated C–H stretching, O–H stretching of the carboxylic group, C=O stretching of the carboxylic group, saturated C–H bending, and C–N stretching in amines.^25,26^ The bands, characteristic of the indole ring, present at 743 cm^–1^, 581 cm^–1^, and 426 cm^–1^ are associated with C–H bending in the aromatic ring and out-of-plane indole ring deformation.^27^

**Figure 4:**
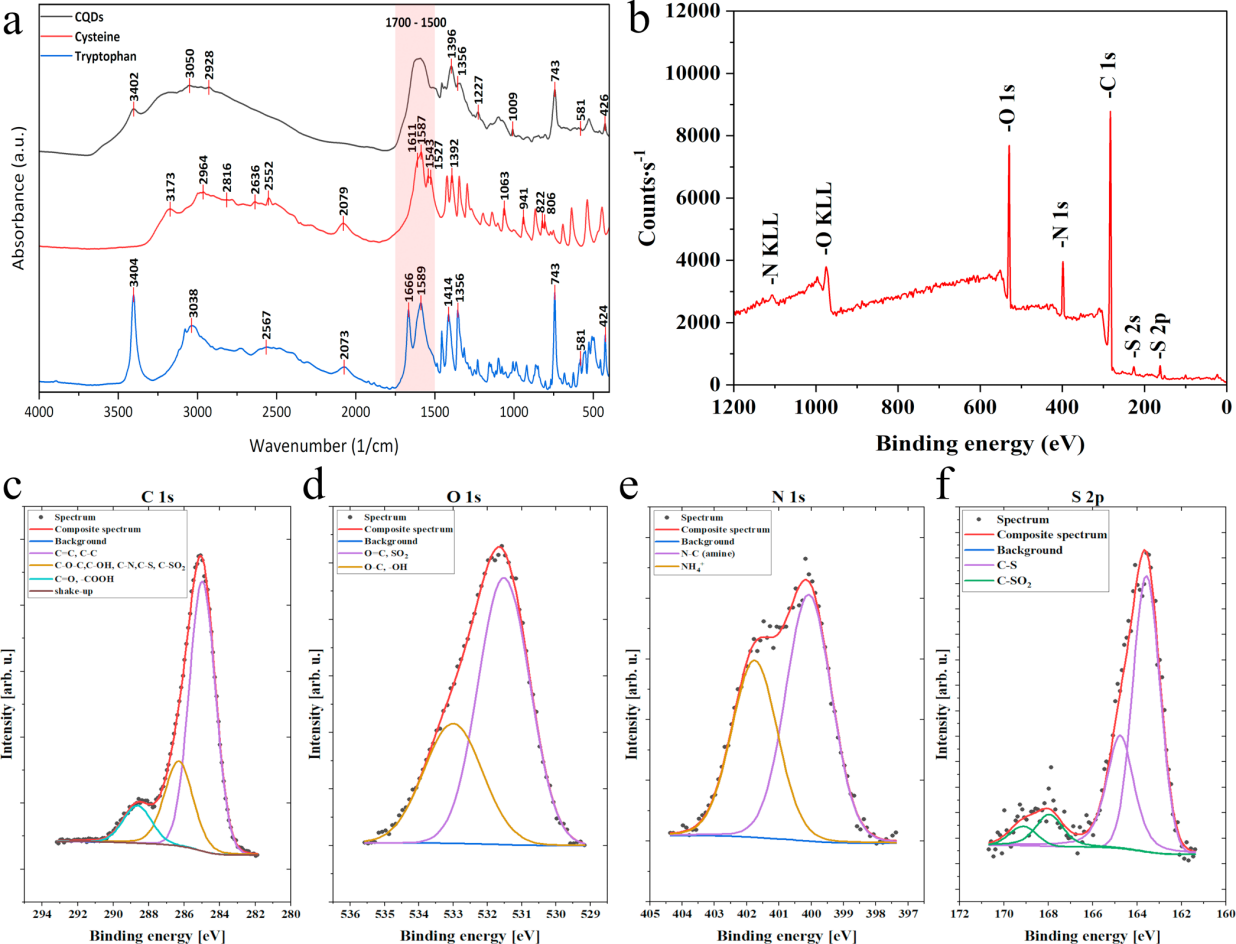
FT-IR spectra of CQDs and precursors (L–cysteine and L–tryptophan) (a). Full XPS spectrum (b); high-resolution XPS spectra of C 1s (c), O 1s (d), N 1s (e), and S 2p (f).

The spectrum of l-cysteine shows the characteristic bands for this amino acid at 2552 cm^–1^ and 941 cm^–1^ associated with S–H stretching and bending vibrations, respectively.^28,29^ The band at 2816 cm^–1^ is indicated on saturated C–H stretching vibration.^14^ The shoulder band at 1610 cm^–1^ and the bands at 1392 cm^–1^, 822 cm^–1^, and 806 cm^–1^ are attributed to asymmetric and symmetric stretching, bending, and wagging of COO^–^, respectively.^29,30^ The bands at 2636 cm^–1^, 2964 cm^–1^, and 3173 cm^–1^ are attributed to NH_3_^+^ stretching, and the bands at 1587 cm^–1^, 1543 cm^–1^, and 1527 cm^–1^ are from NH_3_^+^ bending. The band at 1063 cm^–1^ is associated with NH_3_^+^ rocking.^29^ The absorption band around 2080 cm^–1^, observed for both amino acid crystals (2073 cm^–1^ for l-tryptophan and 2079 cm^–1^ for l-cysteine), may be indicative of extensive H-bonding networks derived from the NH_3_^+^ group. The resulting band is attributed to the combination mode of out-of-plane bending and torsion of the NH_3_^+^ group.^31–33^

In the spectrum of CQDs, the broad band at 3400–3100 cm^–1^ corresponds to the N–H and carboxylic O–H stretching.^25^ The broad band between 1500 and 1700 cm^–1^ covers the range of wavenumbers corresponding to C=C cm^–1^, C=N cm^–1^, and C=O cm^–1^ stretching vibrations indicating the presence of the polycyclic aromatic structure of graphite.^17,34^ The strong bands at 1396 cm^–1^ and 1356 cm^–1^ can be assigned to C–N stretching indicating the presence of the amide III in the structure.^35,36^ The bands at 2928 cm^–1^ and 3050 cm^–1^ are associated with saturated and unsaturated C–H stretching, respectively.^25,37^ For CQDs, all bands derived from the indole ring of tryptophan at 3402 cm^–1^, 743 cm^–1^, 581 cm^–1^, and 426 cm^–1^ are present. This means that it is not fully involved in the formation of the carbon quantum dots core but is also a part of their shell. Moreover, the S–H band is no longer prominent in the CQDs spectrum. Instead, new bands at 1227 cm^–1^ and 1009 cm^–1^ associated with C=S and C–S stretching vibrations, appeared.^38,39^ Furthermore, the band at ∼2080 cm^–1^ disappeared due to the deprotonation of the NH_3_^+^ group of both amino acids.^31^

The presence of the indole ring of tryptophan in the surface state of the carbon quantum dot was confirmed using Raman spectroscopy (see Figure S1 in the Supporting Information). The spectra revealed the dominant nature of the surface states of the CQDs.

The XPS survey spectrum of the N,S-doped CQDs (see Figure 4) revealed the presence of four characteristic C 1s, N 1s, O 1s, and S 2p peaks at 285, 339.1, 530.6, and 163.6 eV, respectively. The C 1s spectra for both samples were fitted with four components. The first line was found at 285.0 eV which could indicate aromatic C=C and/or aliphatic C–C bonds; the second line lies at 286.3 eV and indicates the presence of C–O–C, and/or C–OH, and/or C–N, and/or C–S, and/or C–SO_2_ bonds, the third line positioned at 288.6 eV represents C=O and/or −COOH bonds, and the fourth line at 291.9 eV is attributed to π to π* shakeup satellite. The shakeup excitation originates from the sp^2^ carbon and its aromatic forms and is an additional parameter confirming the presence of this type of bonds.^40,41^ The O 1s spectra were fitted using two components, where the first line was found at 531.5 eV which indicates presence of O=C bonds in organic compounds and/or S–O bonds, and the second line at 533.0 eV originates from either O–C, and/or −OH type bonds, and/or adsorbed H_2_O.^42,43^ The obtained data are consistent with the FT-IR results, confirming the presence of an aromatic, graphite-based core and oxide groups on the surface. The N 1s spectra were fitted with two lines: the first centered at 400.0 eV indicates the presence of C–NH (amine) type bonds, and the second positioned at 401.7 eV comes from NH_4_^+^ type ion presence.^43^ The S 2p spectra were fitted using two doublets (p_3/2_–p_1/2_ doublet splitting is 1.2 eV), in which the first 2p_3/2_ line at 163.6 eV shows the presence of C–SH type bonds and the second 2p_3/2_ line at 167.9 eV represents SO_2_–C species.^44^ These results clearly demonstrate that heteroatoms are present in the structures of CQDs. Surface concentrations of chemical bonds obtained from fitting XPS data for the analyzed samples are listed in Table 2.

**Table 2:** Surface Composition (at%) Determined by Fitting XPS Data

Element (at%)	C				N		O		S	
Binding energy [eV]	285.0	286.3	288.6	291.9	400.0	401.7	531.5	533.0	163.6	167.9
**Species**	**C=C**, C–C	**C–O–C, C–OH, C–N C–S**, **C–SO_2_**	**C=O COOH**	**shakeup**	**N–C**	**NH**_**4**_^**+**^	**O=C**, **C–SO_2_**	**O–C, −OH**	**C–S**	**C–SO**_**2**_
**N,S-doped CQDs**	46.5	16.9	7.3	0.2	5.7	4.1	11.5	6.0	1.2	0.2

The optical properties of the prepared CQDs are presented in Figure 5. The fluorescence emission spectra of CQDs at different excitation wavelengths as well as the 2D excitation–emission map are presented in Figure 5a,b. It could be observed that the shift in the excitation wavelength did not affect the emission wavelength, which remained at 445 nm. This indicates that the obtained CQDs exhibited excitation–unrelated fluorescence properties. The maximum emission intensity was obtained at an excitation wavelength of 380 nm, with a relative quantum yield of 50%. A similar QY (41.3%) was obtained by Yang et al. for nitrogen–containing CQDs obtained by the hydrothermal method.^45^ The CIE spectrum in Figure 5c indicated that this fluorescence is perceived by the human eye as blue in color.^17^ The fluorescence map shows two distinct emission areas associated with the core–shell structure of CQDs (Figure 5b). The first fluorescent center, observed in the range of 300 and 320 nm, indicates the “core state”, whereas the second (350 nm–400 nm) region can be associated with the “surface state” of carbon quantum dots.^46^

**Figure 5:**
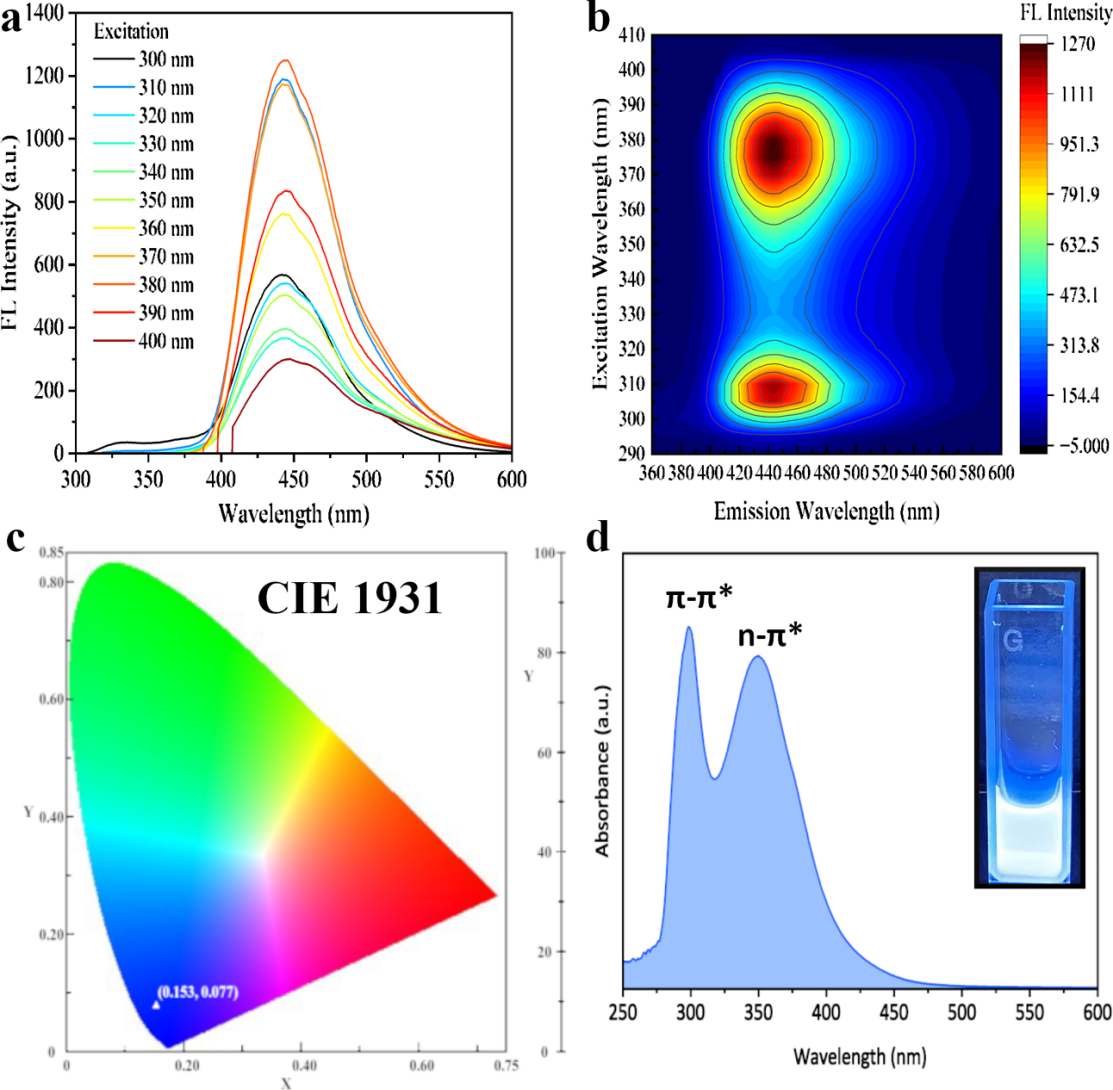
Emission spectra of the obtained CQDs (a); fluorescence excitation–emission map recorded for CQDs (b); CIE spectrum (c). UV–Vis spectrum of CQDs; inset: CQDs solution irradiated with 365 nm wavelength (d).

The UV–Vis spectrum of CQDs (Figure 5d) exhibits two absorption peaks at λ_max1_ = 298 nm and λ_max2_ = 350 nm, both in the UV range. The absorption at λ_max1_ was attributed to π–π* electron transition of aromatic sp^2^ domains of C=C, related to polycyclic aromatic hydrocarbons.^14,47^ In turn, the broad absorption band at λ_max2_ was attributed to the n−π* electron transition of C=O, C–N, and C=S bonds.^47^ The results of UV–Vis observations were in line with those of fluorescence studies. The higher energy band at 298 nm was attributed to the “core state” of the carbon quantum dots (associated with sp^2^ structural defects in the graphitic core). The broad band at 350 nm, extending to higher wavelengths, was considered as the “surface state” and was associated with the presence of functional groups bonded to the core of CQDs.^13,15,46^ The inset in Figure 5d shows the CQD aqueous dispersion under UV (365 nm) irradiation exhibiting strong blue fluorescence which correlates with the obtained CIE spectra (see Figure 5c). According to the literature, the broad absorption band between 300 and 400 nm shows strong fluorescence properties due to the trapping of excited-state energy by the surface state.^47^ These results remain in line with the high quantum yield calculated for the obtained CQDs. The ratio of the fluorescence intensity of the “surface state” to the “core state” is almost equal in the tested samples. This leads to the conclusion that the properties of the tested CQDs were defined by the surface groups of the dots as much as by their core. As the synthesis temperature increased, the number of functional groups on the surface decreased, and the “core state” became dominant (see Figure S2 in the Supporting Information).

### 3.2 Characterization of the CQDs-Modified Nonwoven

The microstructure of the obtained nonwovens is presented in Figure 6. The SEM observations showed the presence of the nanometric fibers with beads-on-string morphology for both investigated variants (reference and CQDs-modified). In the case of the reference PCL/PVP fibers (REF_f), both the core and shell showed a unimodal diameter distribution (see Figure 6a). The average diameter of the core was within a range of 90–260 nm (with an interquartile range of 150–200 nm), while the beads had a size of 380–530 nm at their widest point. The modification of the shell layer by the CQDs (CQDs_f) contributed to decreasing the fiber diameter to the range of 70–160 nm (with an interquartile range of 100–131 nm) (see Figure 6b). The bead part also decreased to the range of 330–460 nm which implied that the presence of CQDs, probably due to increasing surface charge density, led to smaller fiber diameters.^48^ However, the CQDs-modified fibers exhibited a less uniform microstructure, characterized by the presence of diameter outliers.

**Figure 6:**
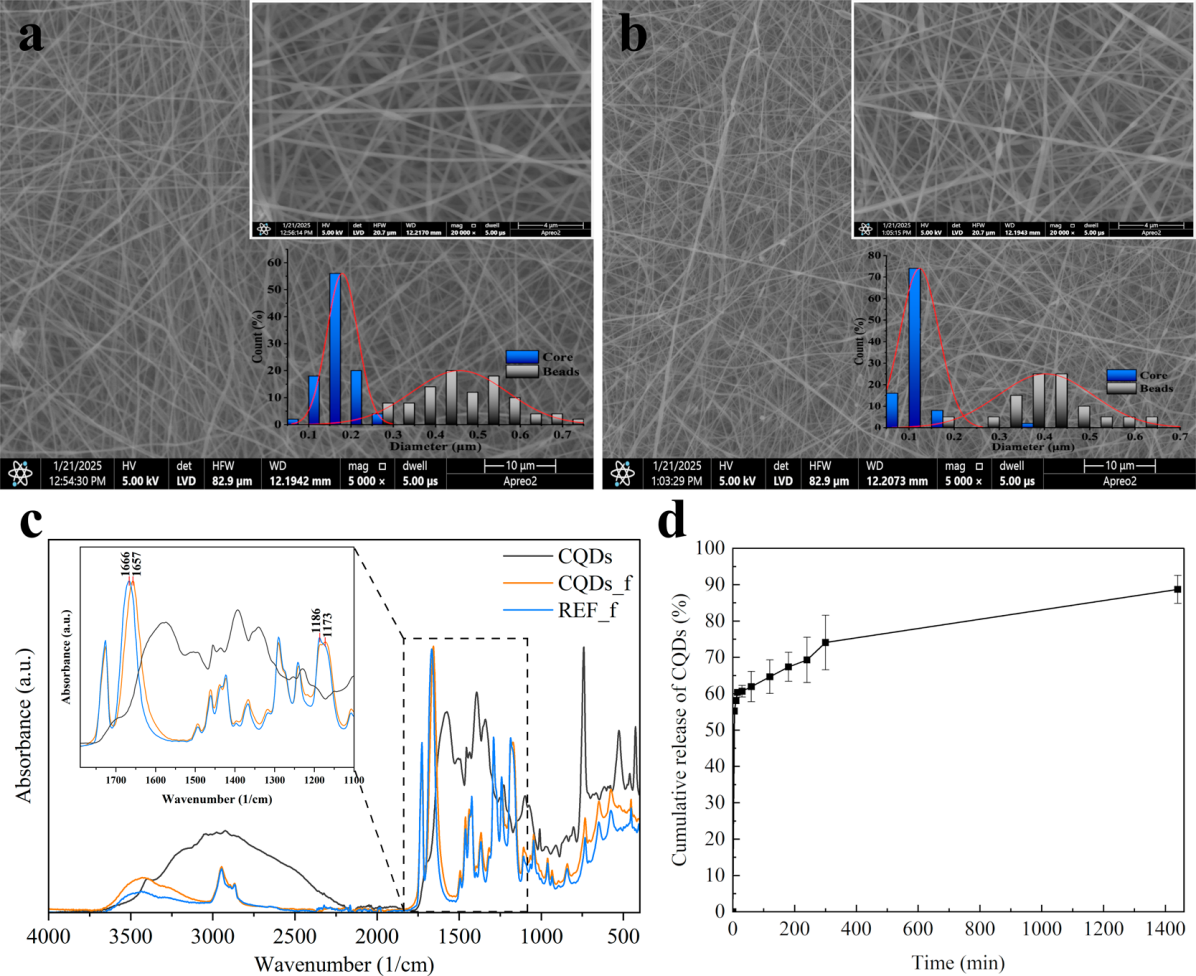
SEM image of the REF_f (a) and CQDs_f (b) samples with fiber diameter size distribution. FT-IR spectra of CQDs-modified fibers (CQDs_f) and PVP/PCL reference spectra (REF_f) (c). Release kinetics of CQDs from the nonwoven (release studies carried out through 24 h) (d).

The FT-IR absorbance spectra of the REF_f and the CQDs_f samples are presented in Figure 6c. Both sample spectra show band characteristic of PCL and PVP. Characteristic bands at 2945 cm^–1^ (CH_2_ asymmetric stretching), 2867 cm^–1^ (CH_2_ symmetric stretching), 1726 cm^–1^ (C=O stretching), 1241 cm^–1^ (C–O–C stretching), and 1186 cm^–1^ (C–O–C stretching) derive from PCL. The bands of PVP at 1290 cm^–1^ and 1494 cm^–1^ are associated with CH_2_ bond twisting and scissor mode, respectively.^49–52^

Further investigation of the CQDs-modified nonwoven material indicated formation of weak interactions between the matrix and the carbon quantum dots, rather than covalent bonds, as no new bands were observed in the FT-IR spectrum. A slight redshift of the C=O bond from 1666 cm^–1^ to 1657 cm^–1^ may indicate a change in the chemical environment caused by hydrogen bonding between PVP and CQDs. Moreover, another redshift of the C–O–C bond from 1186 cm^–1^ to 1173 cm^–1^ suggests that those interactions involve not only the fiber shell but also their core. Besides that, an evident indicator of the presence of carbon quantum dots in the nonwoven is the significant increase in the absorbance of the O–H band (3400 cm^–1^–3100 cm^–1^).

Release kinetics of the CQDs from the nonwoven are presented in Figure 6d. The weak interactions between the additive and the polymer matrix led to the rapid release of 55% of the CQDs within the first 5 min of incubation. Further diffusion into the surrounding medium continued over the next few hours, with the discs used in the antibacterial tests releasing 0.33 mg of CQDs within the first hour and 0.40 mg after 4 h, corresponding to 60–73% of the initial content. Overall, 89% of the CQDs were released after 24 h, with a loading (LE) of 5.8% and encapsulation efficiency (EE) of 80.3%. The nonwoven’s high saturation with the additive and its rapid release ensured the carbon quantum dots’ immediate activity.

### 3.3 Antibacterial Activity Assessment

Performed in the first-place disk-diffusion test of non-illuminated CQDs indicated no zone of bacterial growth inhibition, regardless of the carbon quantum dots suspension concentration. These results indicate that, without light activation, CQDs do not cause a significant reduction in the viability of either *E. coli* or *S. aureus* (see Figure S3 in Supporting Information). The MIC values for CQDs were determined to be 1.25 mg/mL for both *E. coli* and *S. aureus*. Therefore, this concentration was selected for subsequent aPDI assays to ensure relevant and comparable antibacterial evaluation under illuminated and dark conditions.

The results of quantitative measurement of antibacterial activity are presented in Figure 7. Regardless of the incubation conditions (in darkness or illuminated), the *E. coli* strain retained high viability (see Figure 7a). Investigations into the reduction rate of the tested populations (see Figure 7c) revealed that, for pure strains (i.e., incubated without CQDs addition), the percentage of killed bacteria was close to zero. This suggests that illumination alone does not affect *E. coli* viability. The results were completely different when bacteria were exposed to the CQDs. In both groups (illuminated and non-illuminated), a decrease in bacterial viability was observed. However, in the case of the illuminated samples, the reduction of *E. coli* population was much more prominent than for the samples kept in the dark. The contrast between these two variants was visible from the very first hours of the incubation and lasted for the entire 4 h of the study. The most significant difference was obtained for the sample containing the highest concentration of carbon quantum dots. The reduction of the non-illuminated bacteria incubated with the CQDs was within the range of 25–31%, while for the illuminated variant, the reduction was at the level of 89–93%. As the concentration of the carbon quantum dots decreased (CQDs 1/8), the population viability in the dark variant was reduced to ∼40%. For illuminated samples, on the other hand, the bacterial viability reduction rate was approximately 90% for the *E. coli* population.

**Figure 7:**
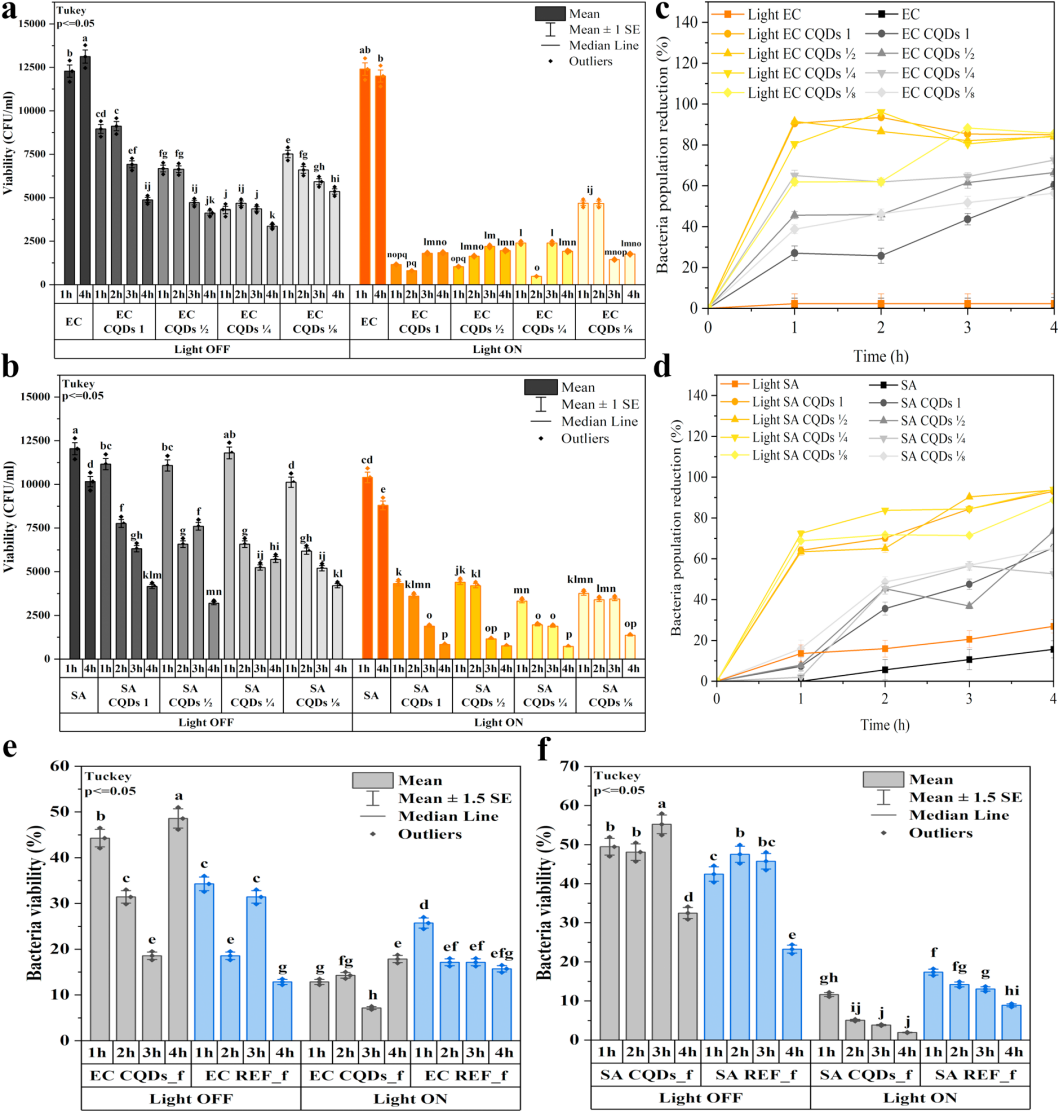
Bacteria viability for illuminated and non-illuminated CQDs incubated with *E. coli* (a) and *S. aureus* (b); reduction in *E. coli* (c) and *S. aureus* (d) populations; bacteria viability for illuminated and non-illuminated CQDs-modified nonwovens incubated with *E. coli* (e) and *S. aureus* (f). *means that do not share a letter are significantly different, SA – *S. aureus*, EC – *E. coli*.

In the case of *S. aureus*, the difference between the illuminated and non-illuminated variants was even more prominent, especially after the first hour of incubation (see Figure 7b). Regardless of the CQD concentration, the reduction in bacterial viability was within the range of 60–80%, while for the variants kept in the dark, more than 90% of the population was still alive (see Figure 7d). Further light exposure resulted in a decrease in the number of viable bacterial colonies, with the rate of reduction depending on the concentration of the CQDs. For higher concentrations (CQDs 1), bacterial viability decreased continuously, whereas for lower concentrations, a sudden drop in viability occurred in the third (CQDs 1/2 and CQDs 1/4) or even fourth hour (CQDs 1/8) of illumination. In contrast to the *E. coli* strain, the viability of *S. aureus* significantly decreased after 4 h of illumination. While the population reduction for *E. coli* was close to zero, it reached 28% for illuminated *S. aureus* samples and 18% for non-illuminated ones. Nevertheless, the CQDs effectively eradicated over 90% of both strains (*E. coli* and *S. aureus*) when concentrations exceeded 125 μg/mL. Representative agar plates illustrating the antibacterial activity of CQDs against *E. coli* and *S. aureus* under illumination are shown in Figures S4–S6 (Supporting Information).

Subsequent studies of bacterial viability in contact with fibrous substrates were conducted to verify whether the carbon quantum dots retain their properties when placed in a polymer matrix (Figure 7e,f). The conducted research showed that the prepared fibers exhibit significantly higher antibacterial activity against *S. aureus*. For this bacterial strain, viability drops to 10% after 4 h of illumination, compared to 30% for non-illuminated bacteria. This value also decreased when the REF_f fibers were irradiated. However, this decrease was significantly smaller than that in the case of fibers modified with the CQDs. In the case of the *E. coli* strain, a decrease in bacterial viability was also observed, although it was not as prominent as in the case of *S. aureus*. The release studies showed that almost 90% of carbon quantum dots were released within the first 4 h. The obtained result may therefore be the effect of the limited diffusion of the CQDs into the bacterial suspension and the higher susceptibility of *S. aureus* bacteria to lower doses of the photosensitizer. Generally, studies of the nonwoven demonstrated high effectiveness of the obtained nanofibrous matrix, modified with the quantum carbon dots, in photodynamic therapy.

### 3.4 Mechanism of Antibacterial Activity

Many studies have indicated the ability to generate ROS as the main mechanism underlying the antibacterial activity of photosensitizers used in aPDI.^15,53^ The quantitative results of intracellular ROS measurements using DCFH-DA are shown in Figure 8. DCFH-DA is a non-fluorescent reagent that forms the fluorescent component DCF after oxidation by intracellular ROS. Increased fluorescence intensity was measured for both control bacterial strains , regardless of whether they were illuminated or not. This suggests that ROS also occurred in pure bacterial strains, regardless of the presence of CQDs. However, this value was significantly higher for bacteria incubated with CQDs that had been exposed to light. For both the illuminated *E. coli* (see Figure 8a) and *S. aureus* (see Figure 8b) strains, the increased fluorescence intensity indicated higher levels of DCFH-DA oxidation and, thus, increased intracellular ROS generation compared to the non-illuminated variant. Moreover, the efficiency of ROS generation was independent of the concentration of CQDs. The fluorescence intensity did not differ significantly for each tested concentration. This suggested that all of the tested variants were effective at generating ROS and exhibited strong antibacterial activity, which was in line with the results of the plate culture test (see Figure 8a,b). Overall, the observed slight increase in DCFH-DA fluorescence suggests that CQDs exhibit low singlet oxygen (^1^O_2_) radical generation and thus limited aPDI activity in the type II pathway (radical generation).^13^ The EPR spectrum obtained for the N,S-modified CQDs is presented in Figure 8c. A quadruplet signal characteristic with equal intensities as well as the spectral parameters (A­(N) ≈ 12.93, A­(H)­β ≈ 10.50, and A­(H)­γ ≈ 1.31) indicates the presence of the DMPO-OOH adduct, suggesting the formation of superoxide (•O_2_^–^) after illumination. The EPR spectrum also shows an additional signal appearing as an additional weak shoulder on the more structured DMPO pattern. This band could correspond to a sulfur-centered radical from S-doped sites, formed simultaneously with superoxide under illumination.^54^ The EPR measurement results suggested that the obtained CQDs are mainly a type I photosensitizer.^55,56^

**Figure 8:**
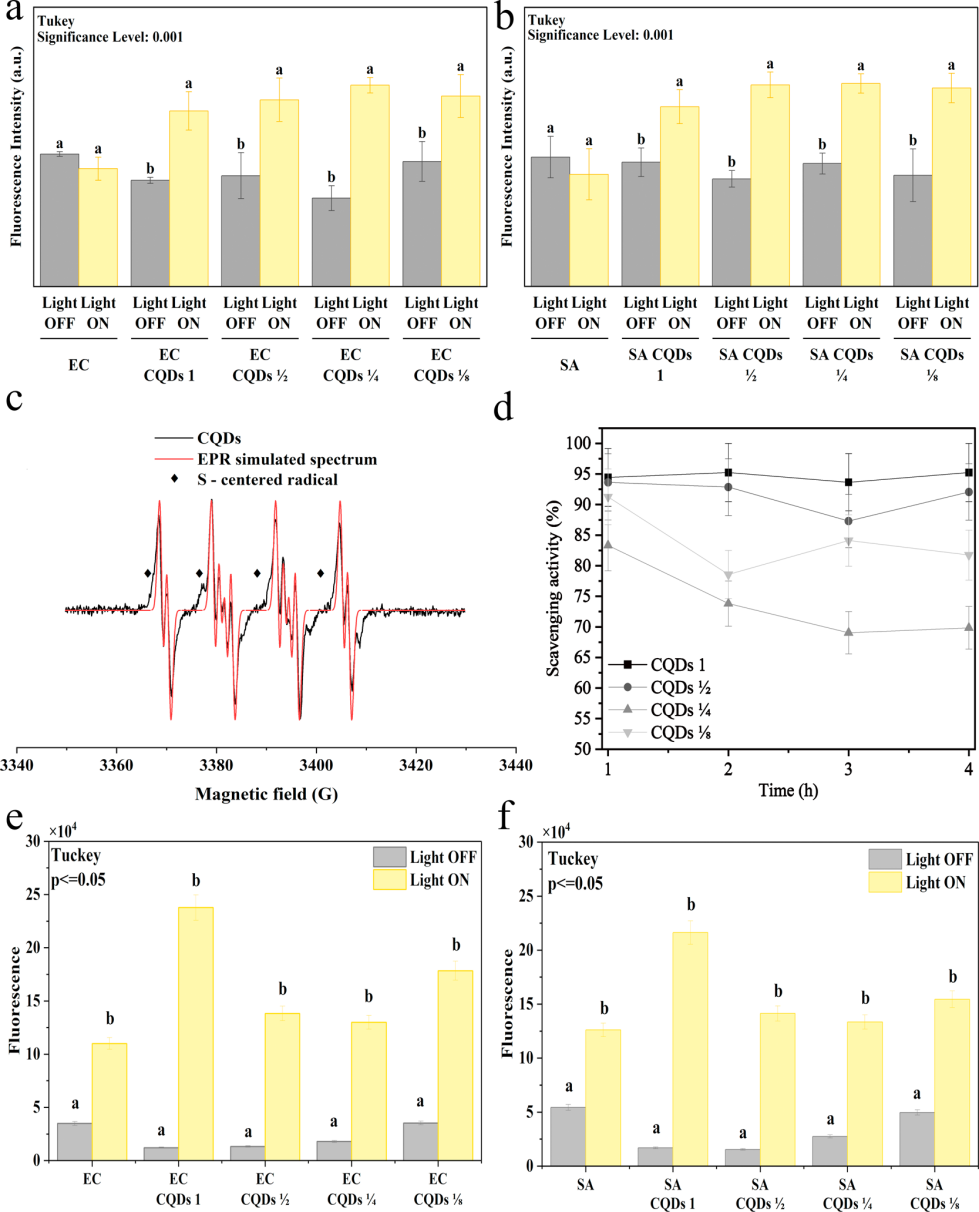
Quantitative measurement of the intracellular ROS generation obtained for *E. coli* (a) and *S. aureus* (b), DMPO EPR signals of •O_2_^–^ generated by CQDs in DMSO under 370 nm laser irradiation (c), and scavenging activity of the non–illuminated CQDs (d). Number of measurements per sample *n* = 3. Scale on the *y*-axis was adjusted between EC (*E. coli*) and SA (*S. aureus*). Membrane permeability measure for *E. coli* (e) and *S. aureus* (f) after 1 h incubation with CQDs. *means that do not share a letter are significantly different.

The radical scavenging activity of CQDs was investigated by using DPPH as a model. The scavenging activity of CQDs at different concentrations is presented in Figure 8d. Interestingly, despite the proven generation of reactive oxygen species, the obtained CQDs also exhibited a high capability for scavenging. The scavenging activity ranged from 70% to 95% and depended on the concentration of the carbon quantum dots. The results of ROS generation and the free radical scavenging measurements indicated the dual nature of the obtained carbon quantum dots, i.e., prooxidant when illuminated and antioxidant in the dark.^15,57^ Similar results were observed by Swain et al. for C,S-doped carbon dots.^58^ Such high antioxidant properties are possible due to the hydrogen transfer from the functional groups of the surface state of the CQDs (−COOH, −OH, −NH_2_). The unpaired electrons on the CQDs surface can be delocalized by resonance within the aromatic domains or through chemical bond rearrangement.^15^ From the obtained results, it can be concluded that the prepared carbon quantum dots are characterized by both anti- and pro-oxidation properties.

Further membrane permeability assays (Figure 8e,f) demonstrated that the investigated CQDs markedly increased the NPN fluorescence intensity in samples containing the highest CQDs concentration under light illumination, irrespective of the bacterial strain tested. Since the NPN probe interacts exclusively with cells exhibiting compromised membrane integrity, these results indicate that illumination-induced activation of CQDs led to enhanced bacterial membrane permeability. Consequently, this effect is expected to potentiate the localized action of ROS generated in close proximity to bacterial cells.

In the mechanistic framework of carbon quantum dots in their emissive excited state, photoexcitation induces ultrafast charge transfer and separation, generating electrons and holes that become trapped at various passivated surface defect sites. These separated charge carriers form highly reactive redox pairs, which under aerobic and aqueous conditions initiate photocatalytic reactions leading to the generation of reactive oxygen species. The specific ROS produced depend on the energy states of the separated electrons and holes.^16^ Therefore, to elucidate the photocatalytic mechanism of CQDs, the energies of the valence band (VB) and conduction band (CB) as well as the band gap energy (*E*_g_) were determined.

The *E*_g_ was determined from Tauc plots. Analysis of these plots demonstrated that all CQDs were more consistently described by the direct allowed transition model (see Figure S7 in Supporting Information). To compare the influence of individual heteroatoms, CQDs synthesized using cysteine (S-containing precursor), tryptophan (N-containing precursor), and a mixture of both precursors were analyzed. The energy gap values determined for the N,S-doped CQDs, tryptophan-based CQDs, and cysteine-based CQDs were 3.14, 4.10, and 3.80 eV, respectively (Figure 9a). On the other hand, the XPS valence band spectra (Figure 9b–d) show that the VB energies of the N,S-doped CQDs, tryptophan-based CQDs, and cysteine-based CQDs are 1.12, 2.92, and 1.45 eV, respectively (corresponding to −7.07, −8.67, and −7.32 eV versus vacuum). By combining these values with the *E*_g_ data obtained from the direct Tauc model, the CB energies were calculated to be −2.08 eV, −1.18 eV, and −2.35 eV (corresponding to −3.87 eV, −4.57 eV, and −3.65 eV vs vacuum) for the N,S-doped, tryptophan-based CQDs, and cysteine-based CQDs, respectively. Based on these results, the potential photocatalytic mechanism was proposed and is illustrated in Figure 9e.

**Figure 9:**
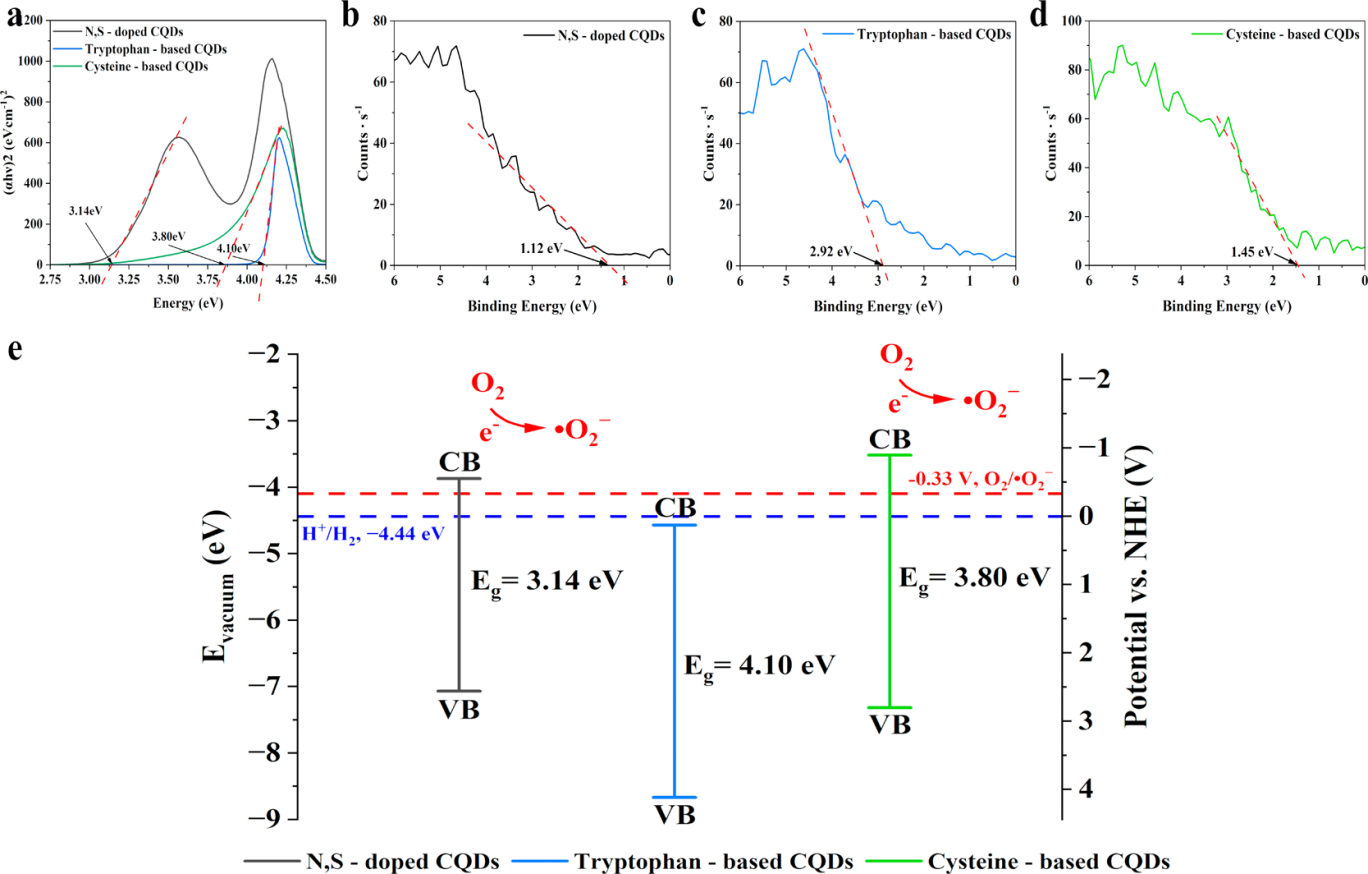
Tauc plot [(α*hν*)^2^ versus *hν*] (a); XPS valence band spectra including valence band (VB) edge of N,S-doped CQDs (b); tryptophan- (c) and cysteine-based (d) CQDs; energy diagram for energy CB and VB edge potentials and charge transfer mechanisms (e). The VB and CB values were determined from XPS valence band spectra and Tauc plots, while the superoxide redox potential versus NHE as well as NHE energy vs vacuum was based on literature data.^16,62^ .

Regardless of the sample variant, the CB edge potentials of the CQDs containing sulfur in their structure (N,S-doped and cysteine-based CQDs) exhibited more negative CB edge potentials compared to the nitrogen-only doped sample (tryptophan-based CQDs). Moreover, these values were more negative than the redox potential of the O_2_/•O_2_^–^ pair (−0.33 V), indicating that sulfur doping facilitates the transfer of photoexcited electrons from the more negative CB to oxygen molecules adsorbed on the CQDs surface, leading to the production of •O_2_^–^ radicals.^16,59^ The obtained results are in good agreement with the EPR data, which identify superoxide as the predominant radical formed in the system. In contrast, the tryptophan-based CQDs exhibited a CB potential more positive than the O_2_/•O_2_•^–^ redox level, which prevents the formation of superoxide under the tested conditions. It is well established that a more negative CB potential favors photoexcited electrons to return to a more positive potential and release more energy. It follows that samples based solely on cysteine show a slightly higher tendency to generate superoxides. However, compared to the other CQDs, N,S-doped CQDs exhibited a notably narrower bandgap (*E*_g_ = 3.14 eV). This bandgap narrowing was attributed to the formation of heteroatom-induced defect states within the carbon network, which facilitated charge transfer between the valence and conduction bands and enhanced photoexcited redox activity.^60^ Furthermore, the reduced bandgap energy was linked to an increased formation of π–π conjugated domains derived from tryptophan, whose presence was confirmed by FT-IR spectroscopy, underscoring their role in modulating the electronic and optical properties of the doped dots. Overall, heteroatom doping—particularly with nitrogen and sulfur—has been widely employed to tune the electronic structure of CQDs, narrow the bandgap, and promote efficient ROS generation under light illumination, rendering them effective photosensitizers for antibacterial and photocatalytic applications.^61^

The antimicrobial behavior mediated by the photosensitizers, besides the ROS generation, can be attributed to either attraction of oppositely charged particles and bacteria (eventually destroying the bacterial membrane structure) or ingestion of the nanoparticles by the bacteria and interaction with its intracellular components leading to programmed death.^63,64^ Moreover, the half-life of ROS, generated during aPDI is on the nanosecond level. Therefore, most of the ROS are quenched before they reach the target bacteria.^53^ This suggests that apart from ROS generation, physical interaction must occur between quantum carbon dots and bacteria. Therefore, to further investigate the mechanism underlying the antibacterial behavior, zeta potential measurements as well as atomic force microscopy were performed.

To check the effect of both CQDs exposure and illumination on the surface charge of bacteria, the zeta potential was measured. The results (see Figure 10) were strongly dependent on the bacterial strain. Regardless of the bacterial strain or the incubation conditions, the zeta potential did not differ significantly in range between illuminated and non-illuminated samples. For *S. aureus* (see Figure 10b), the zeta potential remained in the range −25 mV to −24 mV, and for *E. coli* (see Figure 10a), in the range −11 mV to −13 mV. These results confirmed that illumination had no effect on the viability of the pure bacterial strains, as determined in the plate culture test. For bacteria incubated with CQDs, the zeta potential value over the 4 h test period was within a relatively constant range, regardless of whether the samples were illuminated or non-illuminated. The zeta potential ranged from −10 mV to −20 mV for *E. coli* and from −11 mV to −28 mV for *S. aureus*.

**Figure 10:**
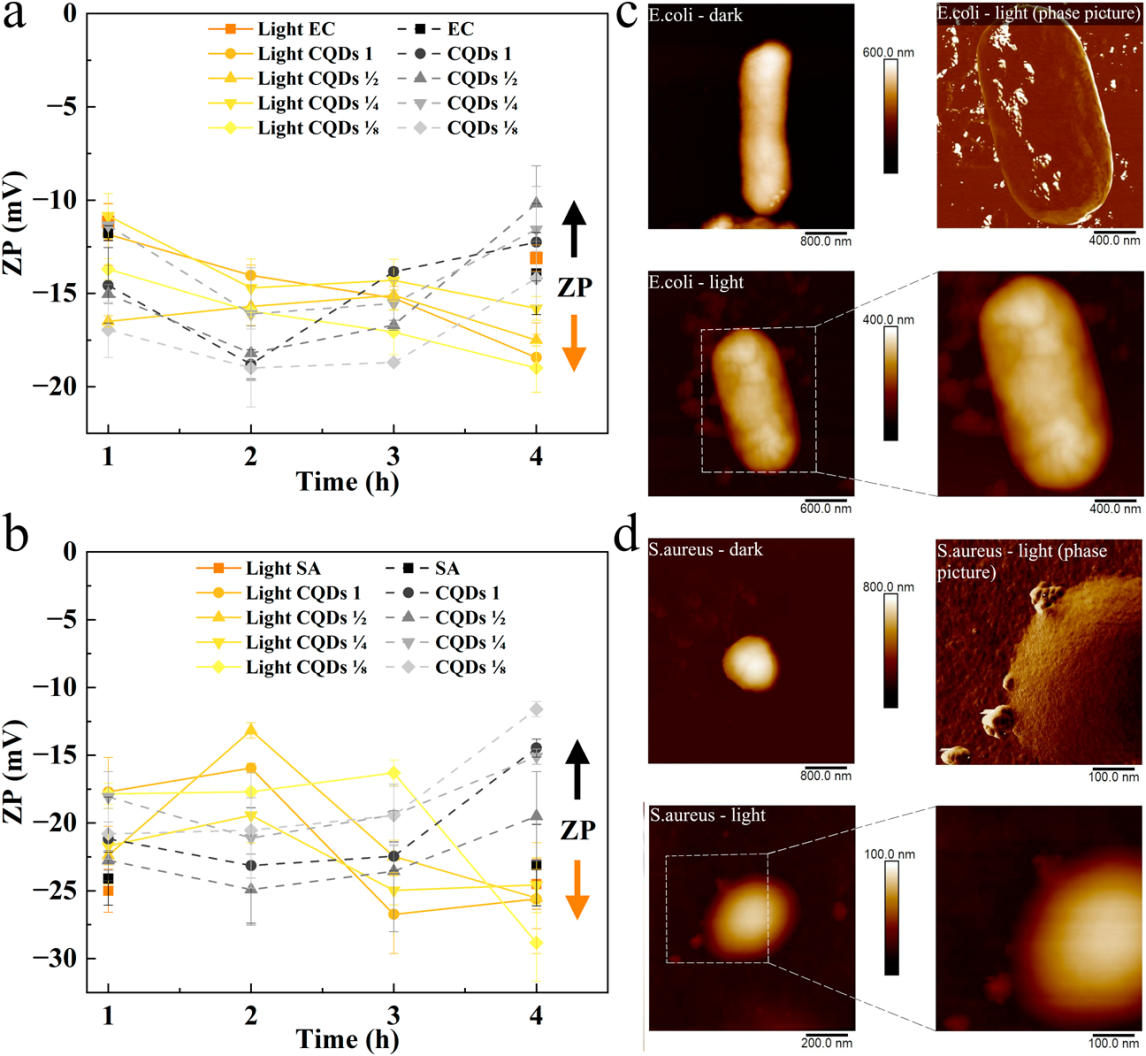
Zeta potential for illuminated and non-illuminated *E. coli* (a) and *S. aureus* (b) (in the presence of the CQDs); AFM observations for illuminated and non-illuminated *E. coli* (c) and *S. aureus* (d) (in the presence of the CQDs). Number of measurements per sample *n* = 3. SA – *S. aureus*, EC – *E. coli*.

However, a common trend of the illuminated versus the non-illuminated samples can be observed. Bacteria subjected to illumination in the presence of CQDs exhibited a decreasing trend in the zeta potential value with duration of illumination, while the non-illuminated samples showed an opposite trend. The identical interaction was observed by Zając et al.^19^ The global zeta potential of *E. coli* and *S. aureus* bacteria is negative, preventing adhesion of negatively charged particles due to the prevailing electrostatic repulsion at the bacteria–nanoparticle interface. However, the bacterial surface is not a homogeneous structure, which, due to the presence of transmembrane proteins, exposes the polar and charged amino acid groups either inside or outside the bacteria. This, in turn, contributes to the presence of local positively or negatively charged sites on the bacterial surface from acidic or basic groups derived from amino acids. The presence of local charged sites on the bacterial surface affects the electrostatic behavior of the bacterial cells and regulates the probability of CQDs adhering to the bacteria. Regarding this, the CQDs can adhere to positively charged sites on the bacterial surface via electrostatic forces and, by that, increase the number of available negatively charged groups (hence the drop of the absolute zeta potential value). Additionally, the attachment of CQDs to bacterial surfaces is facilitated by the presence of surface NH_4_^+^ groups, as confirmed by XPS analysis. This can lead to a reduction in the rate of bacterial growth without necessarily killing the bacteria (as the zeta potential of dead bacteria is less negative than that of living bacteria).^19^ In the current study, the increase in the absolute value of zeta potential was observed only for the illuminated samples, which suggests that the attraction between bacteria and CQDs is triggered by light exposure.

To verify the adopted assumptions about light-triggered adhesion of the CQDs to the bacterial surface, AFM observations were made on bacteria incubated for 4 h in light and in the darkness. The morphology of both tested bacteria is shown in Figure 10c,d. It can be observed that bacteria not exposed to illumination exhibited a correct morphology (*E. coli* *–* Figure 10c) and even some of them were in a division phase (*S. aureus* *–* Figure 10d). The surfaces of both bacteria types were smooth, their dimensions were correct, and most importantly, there were no carbon quantum dots attached to them. The bacterial morphology was completely different for the illuminated variants. The size of the bacteria significantly decreased, probably due to increased membrane permeability and leakage of cytosolic fluids, which is in line with the NPN assay and zeta potential measurement.^36,65^ The bacterial surface was covered with adhered CQDs which incorporated into Gram-positive (*S. aureus* – phase picture) and Gram-negative (*E. coli* – phase picture) bacterial cells, enabling the generation of reactive oxygen species directly inside the bacteria.^53^ Similar results have been obtained by Gagic et al. for biogenic amine-modified carbon quantum dots, where the surface functionalization contributed to the penetration of carbon dots into intracellular media and the subsequent irreparable ROS-induced DNA damage.^66^

### 3.5 Biosafety of the CQDs and Nonwovens *In Vitro*

Fibroblasts play a key role in corneal wound regeneration, participating in processes such as the production of growth factors and chemokines, the differentiation of corneal epithelial cells, and the production of collagenases and metalloproteinases.^67^ Hence, their proper functioning guarantees the success of the corneal regeneration process after implantation. To assess the reaction of human fibroblasts exposed to the CQDs and the obtained nonwovens, viability and toxicity tests were performed. Figure 11 shows the results of the *in vitro* tests after direct contact of the CQDs with BJ cultures. The statistical analysis performed showed high viability of the tested cells exposed to the prepared CQDs, comparable to the reference (TCPS – tissue culture polystyrene) (Figure 11a). The sample with the highest CQDs concentration exhibited a statistically significant difference in cell viability indicating the possible cytotoxic effect due to the detrimental effect of overproduced ROS. However, the sample with a CQD concentration of 0.5 mg/mL showed similar cell viability compared to TCPS, and the sample with a concentration of 0.25 mg/mL showed statistically significantly higher cell viability. Such high cell viability is most likely due to the outstanding scavenging activity of the obtained CQDs. It should also be noted that the volume of the CQDs used in cell studies was 10 times higher than the one used in the bacterial studies. The aim of the *in vitro* test was to determine the critical dose of CQDs causing cytotoxic effects. This means that with the safe doses (CQDs 1/2–CQDs 1/8), it is possible to increase the concentration of carbon dots up to 10 times, thus increasing the effectiveness of the therapy. These results further confirm that the amount of CQDs released from the nonwoven is substantially lower than the concentrations demonstrated to be safe in the cellular assays and is therefore insufficient to adversely affect cell viability. The influence of the surface state of CQDs and the resulting antioxidant properties can be clearly seen when comparing the viability results of fibroblasts incubated with CQDs 160 and 180 °C (see Figure S8 in Supporting Information).

**Figure 11:**
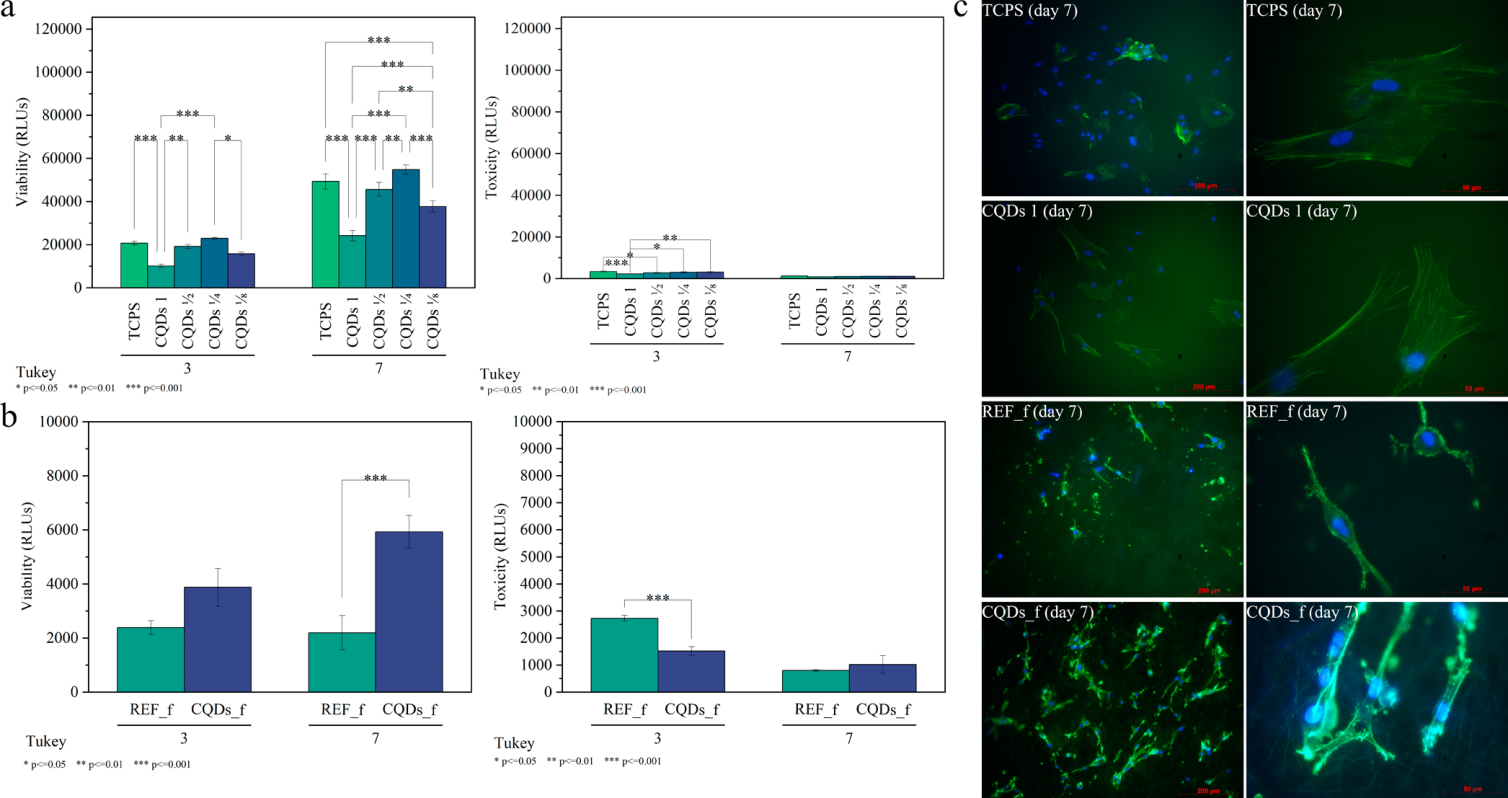
Viability and toxicity of BJ fibroblasts after 3rd and 7th days of incubation with CQDs (a) and fibrous materials CQDs_f, REF (RLUs – relative light units) (b) as well as DAPI-phalloidin stained cells exposed to CQDs 1, CQDs_f, and REF_f (c). TCPS-cultured cells were used as a control (*p ≤ 0.05, **p ≤ 0.01, ***p ≤ 0.001). Fluorescent staining performed after the seventh day of incubation. The charts show results obtained for the whole 1 mL of the CQD suspensions.

Further studies on the CQDs-modified nonwovens (CQDs_f and REF_f) aimed to verify whether encapsulating the carbon quantum dots inside the fiber would allow them to retain their high biological potential. Although the results were not as impressive as those of the carbon quantum dots suspensions, a positive effect of CQDs incorporation on the viability of cells in contact with nonwovens can be observed (see Figure 11b). The presence of the carbon quantum dots contributed to a significant increase in the viability of the BJ cells placed on the surface of the CQDs_f nonwoven.

The positive effect of the quantum carbon dots on cellular viability and morphology *in vitro* is even more evident in images obtained with a fluorescence microscope. Figure 11c shows fibroblasts stained with DAPI and phalloidin dyes. In the case of TCPS and the CQDs suspension, the cells exhibited flat, elongated, and spindle-shaped morphology with a large spreading area. Contrary to the TCPS, fibroblasts placed on a nanofiber substrate tended to occupy the surface more densely. The cells were smaller and thinner but with readily extended filopodia (especially in the case of CQDs-modified fibers).^68^ The observed differences in the cell morphology are in line with previous studies on the interaction of fibroblasts with nanofibers in comparison to films.^69,70^

## 4 Conclusions

In this study, we developed a novel amino acid-derived, N,S-doped carbon quantum dots system exhibiting dual pro- and antioxidant functionality for photoinduced antibacterial therapy and corneal tissue applications. The CQDs were synthesized via a hydrothermal method (160 °C, 6 h) using amino acid precursors and demonstrated a high quantum yield of 50% and strong fluorescence (λ_ex_= 380 nm, λ_em_= 445 nm). Upon light irradiation (390–700 nm, 30 mW/cm^2^), the CQDs generated reactive oxygen species-predominantly superoxide radicals (•O_2_^–^) and a minor fraction of singlet oxygen^1^ O_2_), leading to a reduction of 90% in *E. coli* and 80% in *S. aureus* viability. The antibacterial mechanism involved photoinduced ROS generation combined with electrostatic attachment of the CQDs to bacterial membranes, resulting in membrane disruption and cytoplasmic leakage.

Mechanistic analysis of the N,S-doped CQDs revealed that the heteroatom doping enhances charge separation and ROS generation efficiency, providing a direct link between dopant-induced electronic structure modulation and antibacterial performance. In the absence of light, the CQDs exhibited antioxidant activity of up to 90%, improving human fibroblast cell viability by 35%, thereby demonstrating cytoprotective potential.

When encapsulated within a PCL/PVP electrospun core–shell fibrous matrix, the CQDs maintained their photoactivity while significantly enhancing the overall biocompatibility of the scaffold. The dual redox functionality of the CQDs-mediated antibacterial activity under illumination and antioxidant protection in darkness represents a substantial advancement over conventional single-function photosensitizers. The resulting CQDs-modified nanofibrous platform therefore holds strong promise as a biologically adaptive, light-responsive material for combating antibiotic-resistant ocular infections and supporting corneal regeneration.

## Supplementary Material



## Notes

Raman spectroscopy, further studies of the optical and biological properties; Kirby–Bauer test, plate culture studies results, and Tauc plot (PDF)
