# Supplementary material for: Photodynamic Antibacterial Nanofibers with Tunable Pro- and Antioxidant Activity via N,S-Doped Carbon Quantum Dots for Corneal Tissue Engineering
Source: ACS Appl Mater Interfaces. 2025 Dec 9;17(51):68955–72. doi: 10.1021/acsami.5c16701 (PMC12754744; doi:10.1021/acsami.5c16701)
Supplement: Supplementary file 1 [file am5c16701_si_001.pdf]

Supporting Information

Photodynamic antibacterial nanofibers with tunable pro- and anti-oxidant activity via N,S-doped carbon quantum dots for corneal tissue engineering

Roksana Kurpanik[1], Anna Ścisłowska-Czarnecka[2], Zofia Kucia[3], Agnieszka Lechowska-Liszka[2], Nikola Lenar[4], Agnieszka Różycka[5], Marcin Sarewicz[6], Grzegorz Szewczyk[6], Ewa Stodolak-Zych[1]*

[1] Department of Biomaterials and Composites, Faculty of Materials Science and Ceramics, AGH University of Krakow, 30-059 Krakow, Poland;

[2] Department of Cosmetology, University of Physical Education in Krakow, 31-571 Krakow, Poland;

[3] Department of Silicate Chemistry and Macromolecular Compounds, Faculty of Materials Science and Ceramics, AGH University of Krakow, 30-059 Krakow, Poland;

[4] Department of Analytical Chemistry and Biochemistry, Faculty of Materials Science and Ceramics, AGH University of Krakow, 30-059 Krakow, Poland;

[5] Department of Building Materials Technology, Faculty of Materials Science and Ceramics, AGH University of Krakow, 30-059 Krakow, Poland;

[6] Department of Biophysics, Faculty of Biochemistry, Biophysics and Biotechnology, Jagiellonian University, Krakow, Poland

Corresponding Author

Kurpanik Roksana, kurpanik@agh.edu.pl;

The Raman spectra are presented in Figure S1a. Bands at 759 cm^{-1} and 1010 cm^{-1} are associated with the ring breathing vibrations of the indole ring. The band at 882 cm^{-1} and 1554 $^{cm-1}$ is associated with the H–scissoring on the indole ring and indole ring stretching, respectively. Bands at 1360 cm^{-1} and 1460 cm^{-1} are attributed to CH2-related vibrations. The relatively weak bands at 1078 cm^{-1} and 1340 cm^{-1} are H–scissoring of pyrrole and C–H – bending, respectively. In the region below 759 cm^{-1}, most of the weak bands belong to the deformations of the benzene ring [1]. The fluorescence map shows the distribution of the indole ring within the sample. The regions characterized by different fluorescence intensity are shown in Figure S1b. It indicates the dominant character of the surface state, compared to the core state, as well as the different degree of CQDs surface functionalization.

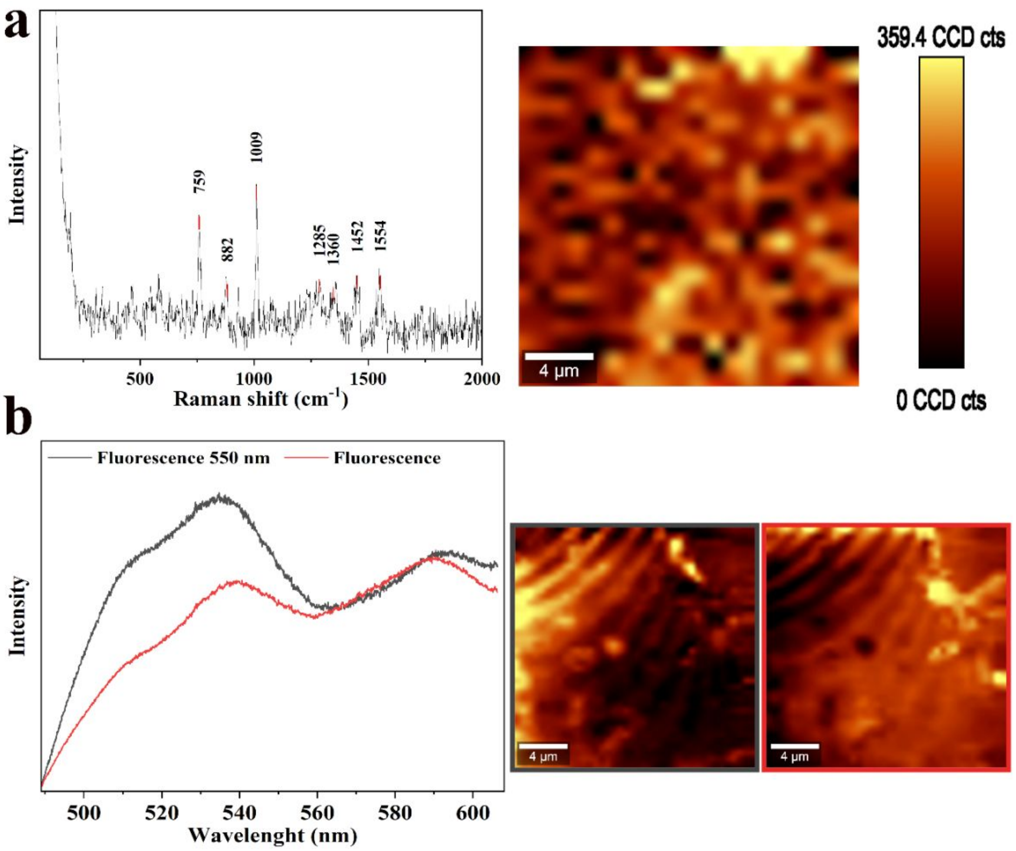

Figure S1 Raman spectrum using 785 nm laser (left) and distribution image of 1009 cm-1 band (right) plus scale (a) and Emission spectra acquired using 488 nm laser (left) and their distributions (right). First – higher emission (black spectrum), latter (red spectrum) (b).

The UV-Vis spectrum of CQDs (see Figure S2) exhibits two absorption peaks corresponding to the CQDs obtained at 180˚C (λ_{max1} = 298 nm and λ_{max2} = 350 nm). However, unlike them, the ratio of core state to surface state is significantly higher. This is due to the increase in the carbon content and the increase in the size of the quantum carbon dots core as a result of progressive carbonization. These dots are characterized by significantly lower photosensitivity in the blue light range [2].

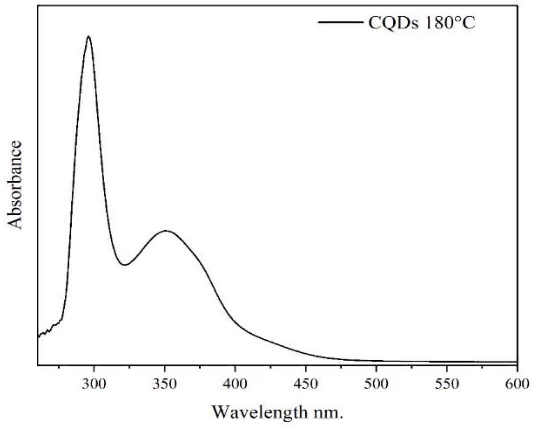

Figure S2 UV- Vis spectrum of CQDs prepared at 180°C.

Figure S3 shows the results of the Kirby-Bauer test for different concentrations of CQD suspensions that were incubated in the dark. For both bacteria, the presence of a clear, confluent layer without growth inhibition zones indicates that non-illuminated carbon quantum dots have no antibacterial activity.

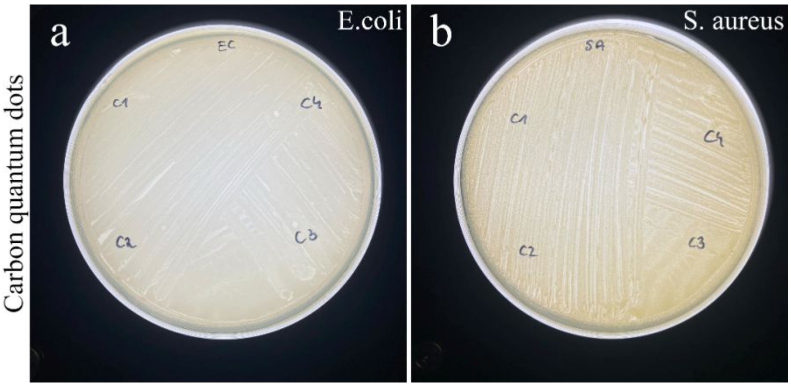

Figure. S 1 The disk-diffusion test results for (a) E.coli (EC) and (b) S.aureus (SA - b) strains. The C1-C4 samples describes different concentration of CQDs (from 1 mg/ml to 0.125 mg/ml).

The photographs of the *E.coli* and *S.aureus* colonies on the agar plates after treatment with various concentrations of CQDs in combination with illumination are presented in Figure S4–S6. The number of bacterial colonies observed on the agar plates is consistent with the quantitative CFU results, confirming the reliability of the antibacterial assessment. In the control samples (without CQDs or illumination), both E. coli and S. aureus exhibited normal

growth, indicating that the experimental conditions did not affect bacterial viability in the absence of treatment.

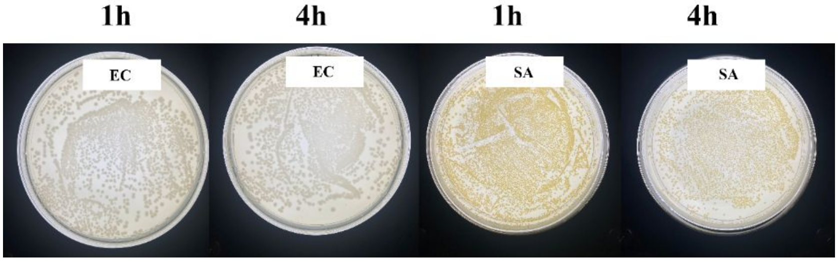

Figure S4 E.coli and S.aureus control colonies after 1h and 4h of incubation.

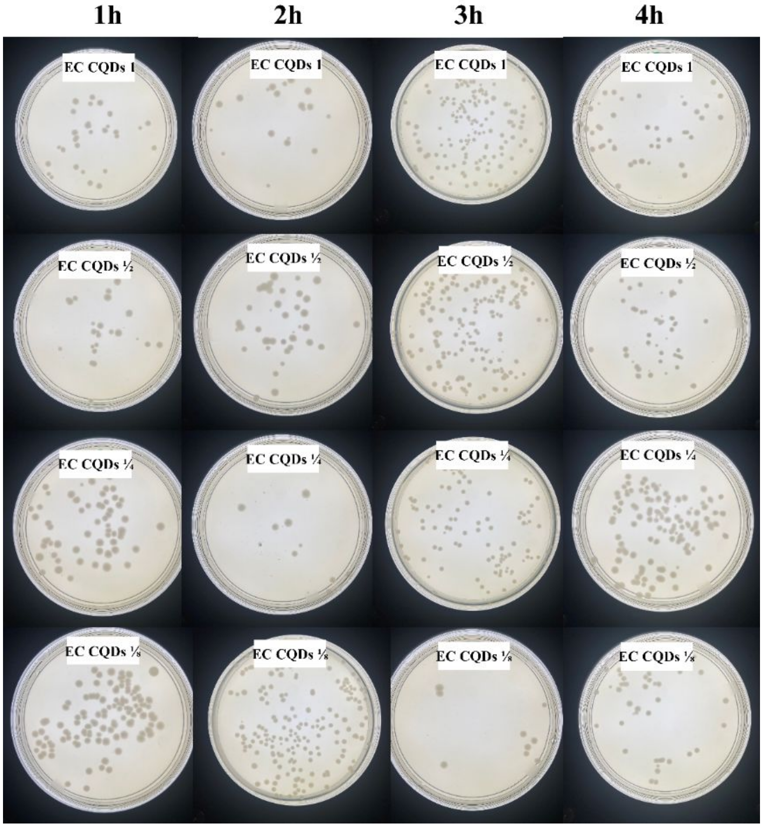

Figure S5 Photographs of E.coli colonies on the agar plates after treatment with various concentrations of CQDs in combination with illumination with a LED diode at a power of 30 mW/cm² for exposures of 1, 2, 3, 4 hours.

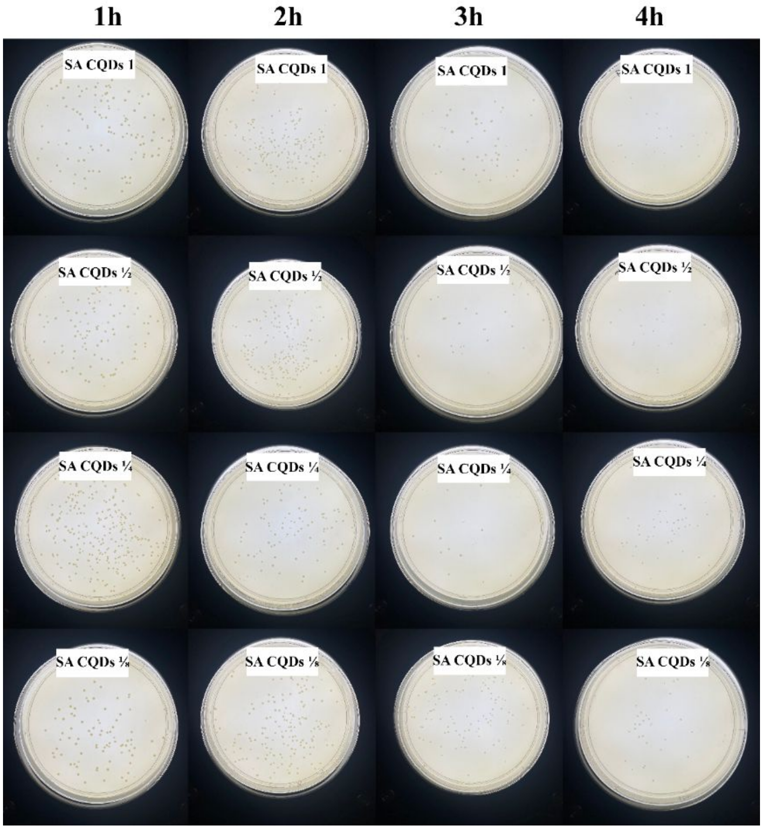

Figure S6 Photographs of S.aureus colonies on the agar plates after treatment with various concentrations of CQDs in combination with illumination with a LED diode at a power of 30 mW/cm^2 for exposures of 1, 2, 3, 4 hours.

Figure S7 presents the Tauc plot obtained for the CQDs. The optical bandgap energies were estimated using both direct and indirect transition models. For the indirect model, the energy gap values for N,S-doped CQDs, tryptophan-based CQDs, and cysteine-based CQDs were 2.80 eV, 3.98 eV, and 3.18 eV, respectively. In the case of the direct model, they were 3.14 eV, 4.10 eV, and 3.80 eV, respectively. Among the two, the direct transition model exhibited a longer linear region and a higher correlation coefficient, indicating better convergence of the experimental data and a more reliable estimate of the optical bandgap.

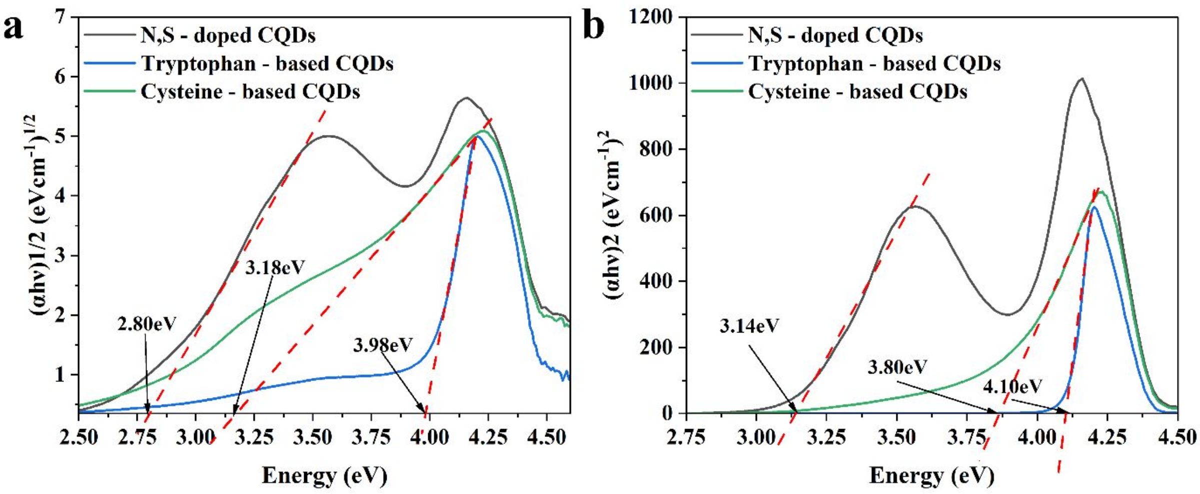

Figure S7 Tauc plot for indirect (a) and direct (b) optical absorption.

The results of in vitro studies on CQDs obtained at 180˚C are shown in Figure S8. In the case of these dots, significantly lower cell viability was observed (more than twice as low for the best variant). This may be due to a reduction in the amount of biologically active functional groups on the surface of CQDs, which probably limits both their ability to form bonds with cells and their antioxidant properties.

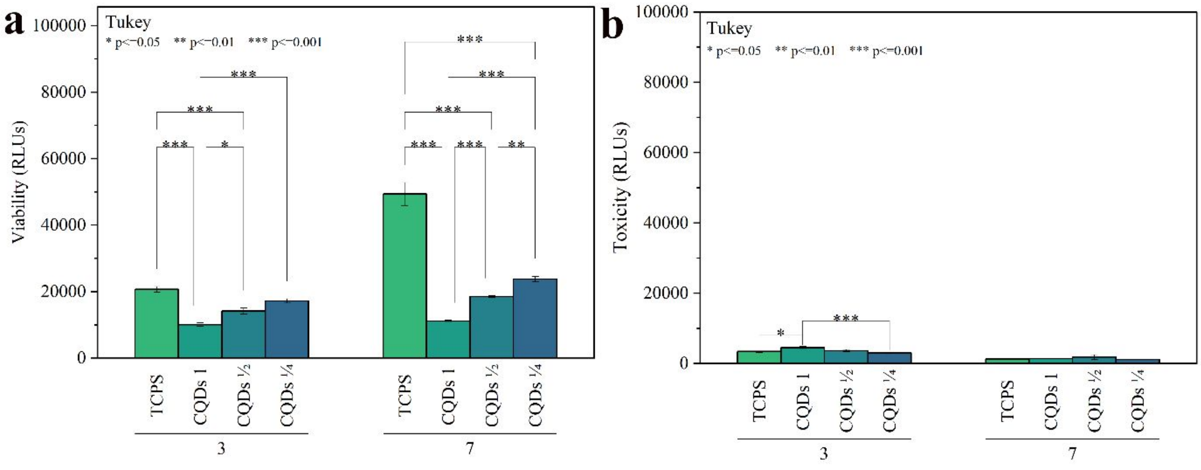

Figure S8 Viability (a) and Toxicity (b) obtained for BJ fibroblasts incubated with CQDs obtained at 180˚C (RLUs – Relative Light Units)

REFERENCES

(1) Zhu, G.; Zhu, X.; Fan, Q.; Wan, X. Raman Spectra of Amino Acids and Their Aqueous Solutions. *Spectrochim. Acta - Part A Mol. Biomol. Spectrosc.* **2011**, *78* (3), 1187–1195. https://doi.org/10.1016/j.saa.2010.12.079.

(2) Yoo, H. J.; Kwak, B. E.; Kim, D. H. Competition of the Roles of π-Conjugated Domain between Emission Center and Quenching Origin in the Photoluminescence of Carbon Dots Depending on the Interparticle Separation. *Carbon N. Y.* **2021**, *183*, 560–570. https://doi.org/10.1016/j.carbon.2021.07.054.
